# Supplementary material for: Plain packaging of waterpipe tobacco? A qualitative analysis exploring waterpipe smokers’ and non-smokers’ responses to enhanced versus existing pictorial health warnings in Egypt
Source: BMJ Open. 2018 Oct 23;8(10):e023496. doi: 10.1136/bmjopen-2018-023496 (PMC6224725; doi:10.1136/bmjopen-2018-023496)
Supplement: Supplementary file 1 [file bmjopen-2018-023496supp001.pdf]

Supplementary

Table 1. Total number of interviews and focus group discussions conducted by demographic profile of participants

	FGDs (10, total participants=80)				IDIs (10)			
	Males (7)		Females (3)		Males (7)		Females (3)	
Age	S	NS	S	NS	S	NS	S	NS
	(5)	(2)	(2)	(1)	(6)	(1)	(2)	(1)
18-24	2	1	1		3	1	1	
25+	3	1	1	1	3		1	1

FGDs=Focus group discussions, IDIs=In-depth interviews, S=Waterpipe smoker, NS=Non-smoker

Table 2. Sociodemographic characteristics and waterpipe smoking status of participants

	N=90	%
**Gender**		
Male	**65**	**72.2**
Female	**25**	**27.8**
**Age (range 18-73)**		
**Mean age ± standard deviation** 33.4 ± 11.6		
**Age group, years**		
18-24	**21**	**23.3**
25+	**69**	**76.7**
**Education level**		
Primary or lower	**16**	**17.7**
Secondary	**32**	**35.6**
Tertiary	**42**	**46.7**
**Occupation**		
Employed professional	**42**	**46.7**
Employed non-professional	**34**	**37.8**
In further study or seeking employment	**14**	**15.5**
**Residence**		
Rural	**20**	**22.2**
Semi-urban	**51**	**56.7**
Urban	**19**	**21.2**
**Smoking status**		
Waterpipe smoker	**62**	**68.9**
Non-smoker	**28**	**31.1**
**Ever noticed PHWs on WTPs**		
Yes	**53**	**58.9**
**Where**		
At cafés	**17**	**32.1**
At home	**11**	**20.8**
Buying tobacco	**25**	**47.2**

Interview guide *

**Interview guide questions**	**Parts relevant to this article**
**Introductory questions**<br>*In general, what do you think of waterpipe smoking?*<br>**Personal experiences of waterpipe smoking**<br>*Tell me about your waterpipe smoking, habits, duration, etc…Tell us more on how did you start smoking?*<br>*Could you describe the setting in which you smoked waterpipe last time?*	
**Awareness of pictorial health warnings**<br>*Have you ever noticed the pictorial health warnings on waterpipe tobacco packs?*<br>*Where did you see them? Can you tell us more about the setting?*<br>*How do they look like?*	√
**Positive and negative characteristics of the current/alternative set of PHW**<br>*Have you ever come across the warning on this pack before? What is your first impression about it?*<br>*What do you think/feel when you see it?*<br>*In your opinion, what is good and is bad about it? Why do you think so?*<br>*Do you think it is/would be effective? Why and how do you think so?*	√
**Reactions to placing PHW on the waterpipe device**<br>*What is your first impression about putting the warning in this spot?*<br>*What do you think/feel when you see there?*<br>*In your opinion, what is good and is bad about this spot? Why do you think so?*<br>*Do you think people will notice the PHW on this spot?*<br>*Would it be effective? Why and how do you think so?*	

***This guide contains core questions. Probing questions were used when necessary (examples include questions on salience, affective reactions, perceived harm, credibility)**

Table 3. Themes and subthemes identified from focus groups and in-depth interviews*

**Themes**	**Subthemes**	**Themes covered in this article**
General perceptions of waterpipe smoking	Factors contributing to WTS<br>Factors affecting WTS initiation and continuation<br>WTS setting<br>Patterns of WTS consumption<br>Social and cultural context of WTS<br>WTS compared to cigarette smoking<br>Tobacco product characteristics<br>Perceived health consequences<br>Reasons for quitting	
Views on pictorial health warning labels on waterpipe tobacco packs	General knowledge /awareness of PHWs on WTPs<br>Sources of awareness<br>Most salient/recalled labels, reasons<br>Cessation helpline<br>Perception of PHWs on WTPs motivating and discouraging factors related to viewing PHW labels	Partly
Perceptions of existing waterpipe tobacco warnings	General impressions, attitude (appeal, affective reactions, avoidance, wear out)<br>Positive and negative characteristics of design and content<br>Participants views of the most effective one in general<br>Possible effects on quitting and/or initiating WTS	√
Insights into novel waterpipe tobacco warnings	General impressions, attitude (appeal, affective reactions, avoidance)<br>Positive and negative characteristics of design and content<br>Participants views of the most effective one in general<br>Possible effects on quitting and/or initiating WTS	√
Avenues for development	Participant suggestions of improved label characteristics regarding content, design, location<br>Participant suggestions for other placement locations	Partly
Best location for inserting labels on the waterpipe device and accessories	Views behind choosing each location<br>Concerns about each location<br>Possible effects on quitting and/or initiating WTS	
Participant suggestions for reducing tobacco use in general and WTS in particular	Further strategies for improved regulations<br>Enforcing existing laws<br>Posting warnings in public and other places<br>Associate warnings with mass-media anti-tobacco campaigns, increase taxation and other suggestions	

*** Themes presented in this article were synthesized in respect to warning content and pack design and their perceived likely effect on uptake or cessation of waterpipe smoking, comparing existing versus novel warnings.**

Table 4. Selected participants' quotes representing views of the current and alternative pictorial health warning labels on waterpipe packs. Quotes are presented by gender, age group*, smoking status, and region of participants

Warning label content – perceived likely effect of existing and novel PHWs	
"The picture of a bent cigarette (i.e. representing sexual dysfunction) caught people's attention considerably…but the other warning…people got used to them after a while"	*(Male smoker, >25y, rural, FGD)*
"The most commonly known warning is that of the lungs"	*(Male smoker,>25y, semi-urban, FGD)*
"The picture is disgusting and frightening…"	*(Male non-smoker, <25y, rural, IDI)*
"Pictures are scary and unpleasant"	*(Male smoker, >25y, semi-urban, FGD)*
"The pictures make me sick…"	*(Male smoker, >25y, urban, IDI)*
"The warning shouldn't be that it causes cancer and that's it…that is just routine talk"	*(Male non-smoker, >25y, urban, FGD)*
"I just don't believe it…nobody reaches this stage"	*(Female smoker, <25y, urban, IDI)*
"I don't believe the warnings…I know smoking is harmful but not to that extent. This is unrealistic"	*(Male smoker, <25y, rural, FGD)*
"We want to see the normal lung beside the diseased one…but this one straight is not clear"	*(Male non-smoker, >25y, urban, FGD)*
"The pictures are not realistic, we never see such things in real life"	*(Male non-smoker, >25y, semi-urban, FGD)*
"The pictures are extremely exaggerated"	*(Male smoker, <25y, urban, IDI)*
"Is this a burnt piece of pizza? (face cancer warning)…this one won't be clear to illiterates…I didn't get it before reading the text beneath"	*(Male non-smoker, >25y, urban, FGD)*
"The picture is frightening and is very disgusting"	*(Male smoker, >25y, urban, IDI)*
"It can make me want to look closely to see what the illness shown is"	*(Female non-smoker, >25y, semi-urban, FGD)*
"For me, the most effective one is picture of teeth affection…it's very scary and upsetting. I see that content of the warning is much more important than size"	*(Male smoker, >25y, semi-urban, FGD)*
"With this shape (deformed teeth and gums) I may disturb my son when I come close to kiss him...now that will hurt me"	*(Male smoker, >25y, semi-urban, IDI)*
"The baby's picture may affect women more than me"	*(Male smoker, >25y, semi-urban, IDI)*
"If a pregnant woman saw this picture…she would fear for her unborn child to have anomalies"	*(Female smoker, >25y, semi-urban, FGD)*
"Explain the components of tobacco as glycerin and the hazards of each of them…tell them: try to smoke each component by itself and see what happens to you"	*(Male non-smoker, >25y, semi-urban, FGD)*
"Simplify the matter of quitting to people…especially those who have been smoking for a long time…"	*(Male nonsmoker, >25 y, rural, FGD)*
"If you talk to a smoker about their health…they wouldn't pay attention but there are other aspects as hazards to their children"	*(Male non-smoker,>25y, semi-urban, FGD)*
"The most effective thing that makes a smoker quit is that they themselves get ill or they see someone get ill"	*(Male smoker, >25y, semi-urban, IDI)*
" I saw on the internet a pack of cigarettes with a picture of a mother and	*(Female smoker, <25 y, urban, IDI)*

her child, and on opening it the mother is separated from him. This a new effective idea."	
“A pregnant woman or a mother may ask their smoker husband to stop”	*(Female non-smoker, >25y, semi-urban, FGD)*
"The only thing that can force me to stop smoking, even if I'm a heavy smoker is the baby affection"	*(Female smoker, <25 y, urban, IDI)*
“Warnings should target young people under 17 before they attempt smoking”	*(Male non-smoker, >25y, semi-urban, FGD)*
"Warnings concerning sexual dysfunction can affect young smokers”	*(Male non-smoker,>25y, semi-urban, FGD)*
”Focus on the damage that the family suffers…there are problems that can occur with the wife because of smoking…so smoking may endanger the family bonds”	*(Male smoker, >25y, semi-urban, IDI)*
“Pictures should be more realistic, and not only focus on severe side effects like cancers… like if warnings demonstrate just a fingernail affected due to smoking not all the hand, people will be more tempted to believe it"	*(Male smoker, <25 year, rural, IDI)*
Design Features of WTPs – perceived likely effect of existing and novel PHWs	
“The apples got my attention”	*(Female smoker, >25y, semi-urban, IDI)*
“After seeing this…(pointing to the fruit depicted on the pack)…it will be hard for me to link the idea with the damage in this picture”	*(Male non-smoker, <25y, rural, FGD)*
"Pictures of fruits are attractive and appetizing”	*(Male smoker, >25y, semi-urban, FGD)*
“If there is no flavored tobacco, I will quit"	*(Male smoker, <25y, urban, IDI)*
“I can’t see any flavors on it…I want to hold it closely to know what did they put there instead…if shisha is served as it is…without any flavors?!...I’ll quit”	*(Male smoker, <25y, urban, FGD)*
"If flavors were banned may be some will quit but warnings have no effect”	*(Male smoker, <25y, urban, FGD)*
"The pictures of fruits must be removed as they are appetizing"	*(Male non-smoker, <25y, rural, IDI)*
"The dark background makes the writings clearer”	*(Male non-smoker,>25y, semi-urban, FGD)*
"The alternative pack is clearer regarding the color, the picture and the way of writing"	*(Male non-smoker, < 25y, rural, IDI)*
"The black color makes the pack a lot chic"	*(Male smoker, <25y, urban, FGD)*
"The color of the package is dark and it conveys a sense of pessimism"	*(Male smoker, >25y, rural, IDI)*
"The bigger the picture, the better”	*(Male smoker, <25y, semi-urban, FGD)*
"The warning should be put on top because the pictures of fruits are drawing attention away from the warning”	*(Female non-smoker, >25y, semi-urban, FGD)*
"Oh! I didn’t realize there was a quit line on the pack before…this dark background made some kind of contrast… the text now is way clearer for me to read”	*(Female smoker, <25y, urban, IDI)*
“I used to think about the hazards a lot when the pictures first appeared…then I got used to them…I don’t pay them attention anymore”	*(Male smoker, <25y, semi-urban, IDI)*

"I have seen the warnings too often that they no longer frighten me”	*(Male smoker, <25y, rural, FGD)*
"The pictures are horrible but people got used to them, if a nonsmoker wished to try and saw the warnings they may change their mind but for those who are already smokers, the warnings make no difference	” *(Male non-smoker,>25y, semi-urban, FGD)*
“Warnings influence the non-smokers much more than smokers, as non-smokers usually find the picture more disgusting”	*(Female, non-smoker, >25y, semi-urban, FGD)*
"I avoid buying the picture of the tongue in particular (referring to the mouth cancer warning)”	*(Male smoker, <25y, semi-urban, FGD)*
"People can go around all day looking for a certain picture (the lungs)"	*(Male smoker, >25y, rural, IDI)*
"Vendors used to sell the pack with free stickers to cover the warnings”	*(Male smoker,>25y, semi-urban, FGD)*
"No one would buy a pack with such warning (means warning picturing effect of smoking on children)”	*(Female smoker, >25y, semi-urban, IDI)*
"If the warning on the pack looks bad, we tend to throw the external package away and put tobacco in another package or may be order a certain warning”	*(Male smoker, >25y, rural, FGD)*
"The alternative pack may make some smokers reduce smoking”	*(Male non-smoker, <25y, rural, FGD)*
"A beginner can be affected compared to a person who has been smoking for 15 years and nothing happened to them as a result of smoking”	*(Male non-smoker, <25y, rural, FGD)*
“Non-smokers will be more affected by it, smokers won't be much affected”	*(Female non-smoker, >25y, semi-urban, IDI)*
"It could affect those who want to start smoking, but the older smokers won't be affected much"	*(Male smoker, <25y, semi-urban, FGD)*

***The age group of participants was categorized as > or < 25 years, because we were interested in highlighting the views of young adults who may be at higher risk of experiencing WTS or maybe non-established WT users yet, versus older adults who might more likely have established a smoking or non-smoking behaviour.**
