# Supplementary material for: Plain packaging of waterpipe tobacco? A qualitative analysis exploring waterpipe smokers’ and non-smokers’ responses to enhanced versus existing pictorial health warnings in Egypt
Source: BMJ Open. 2018 Oct 23;8(10):e023496. doi: 10.1136/bmjopen-2018-023496 (PMC6224725; doi:10.1136/bmjopen-2018-023496)
Supplement: Author's manuscript [file bmjopen-2018-023496.draft_revisions.pdf]

BMJ Open is committed to open peer review. As part of this commitment we make the peer review history of every article we publish publicly available.

When an article is published we post the peer reviewers' comments and the authors' responses online. We also post the versions of the paper that were used during peer review. These are the versions that the peer review comments apply to.

The versions of the paper that follow are the versions that were submitted during the peer review process. They are not the versions of record or the final published versions. They should not be cited or distributed as the published version of this manuscript.

BMJ Open is an open access journal and the full, final, typeset and author-corrected version of record of the manuscript is available on our site with no access controls, subscription charges or pay-per-view fees (http://bmjopen.bmj.com).

If you have any questions on BMJ Open's open peer review process please email info.bmjopen@bmj.com

BMJ Open

Plain Packaging of Waterpipe Tobacco? A Qualitative Analysis Exploring Waterpipe Smokers' and Non-smokers' Responses to Enhanced versus Existing Pictorial Health Warnings in Egypt

Journal:	*BMJ Open*
Manuscript ID	bmjopen-2018-023496
Article Type:	Research
Date Submitted by the Author:	09-Apr-2018

Complete List of Authors:	Mostafa, Aya; Faculty of Medicine, Ain Shams University, Department of Community, Environmental, and Occupational Medicine<br>Mohammed, Heba Tallah; Faculty of Medicine, Ain Shams University, Department of Community, Environmental, and Occupational Medicine; University of Waterloo, Ontario, School of Pharmacy<br>Mohamed, Wafaa; Faculty of Medicine, Ain Shams University, Department of Community, Environmental, and Occupational Medicine<br>Elhabiby, Mahmoud; Faculty of Medicine, Ain Shams University, Department of Psychiatry Medicine<br>Safwat, Wael; Egyptian Tobacco Control Coalition<br>Labib, Sahar; Tobacco Control Unit, Ministry of Health<br>Abou-El-Fetouh, Aisha; Faculty of Medicine, Ain Shams University, Department of Community, Environmental, and Occupational Medicine<br>Hoek, Janet; University of Otago, Marketing

Keywords:	Plain packaging, Health warnings, Waterpipe tobacco, Uptake, Cessation, QUALITATIVE RESEARCH

SCHOLARONE™ Manuscripts

RESEARCH ARTICLE

Plain Packaging of Waterpipe Tobacco? A Qualitative Analysis Exploring Waterpipe Smokers' and Non-smokers' Responses to Enhanced versus Existing Pictorial Health Warnings in Egypt

Aya Mostafa[1], Heba Tallah Mohammed[1, 2], Wafaa Mohamed[1], Mahmoud Elhabiby[3], Wael Safwat[4], Sahar Labib[5], Aisha Aboulfotouh[1], Janet Hoek[6]

[1] Department of Community, Environmental, and Occupational Medicine, Faculty of Medicine, Ain Shams University, Cairo, Egypt

[2] School of Pharmacy, University of Waterloo, Ontario, Canada

[3] Department of Psychiatry Medicine, Faculty of Medicine, Ain Shams University, Cairo, Egypt

[4] Egyptian Tobacco Control Coalition, Cairo, Egypt

[5] Tobacco Control Unit, Ministry of Health, Cairo, Egypt

[6] Departments of Public Health and Marketing, University of Otago, New Zealand

CORRESPONDING AUTHOR

Dr. Aya Mostafa (ORCID: http://orcid.org/0000-0002-2803-2608; Scopus ID: 36010130700)

Department of Community, Environmental, and Occupational Medicine, Faculty of Medicine, Ain Shams University, Abbasia Square, Cairo, Egypt, PO-box 11566 Cairo, Egypt

E-mail: aya.kamaleldin@med.asu.edu.eg Phone: +2 0100 5217379

WORD COUNT Abstract 291, Main text 4,092 (Introduction 720, Methods 1101, Results 1402, Discussion 870).

This manuscript has not been published in another journal, nor is it under review elsewhere.

For peer review only - http://bmjopen.bmj.com/site/about/guidelines.xhtml

ABBREVIATIONS

FCTC — Framework Convention on Tobacco Control

PHWs — Pictorial Health Warnings

WHO — World Health Organization

WT — Waterpipe Tobacco

WTS —Waterpipe Tobacco Smoking

WTPs — Waterpipe Tobacco Packs

For peer review only

For peer review only - http://bmjopen.bmj.com/site/about/guidelines.xhtml

ABSTRACT

Objectives Despite the global increase in waterpipe tobacco smoking (WTS), few studies have assessed the effectiveness of waterpipe tobacco (WT) health warnings. Although Egypt has used four generic pictorial health warnings (PHWs) on waterpipe tobacco packs (WTPs) and has rotated these every two years since 2008, WTS rates have continued to rise. We explored in this qualitative study how participants perceived existing PHWs on WTPs, assessed how they interpreted novel plain packaging of WT featuring enhanced PHWs, and probed perceptions of how existing and novel sets would affect uptake or cessation of WTS.

Design We conducted ten qualitative focus groups and ten in-depth interviews. We explored participants' views of the four existing PHWs (occupied 50% of the front and back of WTPs, displayed cancers, and featured colourful fruits and flavors) and four novel PHWs (occupied 80% of the front and back of WTPs, displayed different topical content, with plain packaging). Transcripts were analyzed using thematic analysis.

Setting Rural Menoufia, urban and semi-urban Cairo, Egypt.

Participants 90 waterpipe smokers and non-smokers, males and females, aged 18 years or older.

Outcomes Perceived potential effect on WTS uptake or cessation, probing factors related to PHW content and WTP design.

Results Participants in focus groups and in-depth interviews thought existing WT PHWs elicited affective responses, but found them unclear or unrealistic and thought the colourful packaging detracted from the warnings. In contrast, they thought novel and larger WT PHWs presented in plain packaging might prevent WTS initiation or trigger quit attempts. Participants regarded warnings featuring proximal health risks as most likely to be acceptable.

Conclusions Our exploratory study suggests larger WT PHWs featuring proximal risks and

For peer review only - http://bmjopen.bmj.com/site/about/guidelines.xhtml

presented on plain WTPs could potentially deter experimentation with WT products among non-users and promote cessation among existing users.

The abstract of this study has been presented at the World Conference on Tobacco and Health, 2018 in South Africa: https://doi.org/10.18332/tid/84640.

For peer review only

For peer review only - http://bmjopen.bmj.com/site/about/guidelines.xhtml

ARTICLE SUMMARY

Strengths and limitations of this study

- This is the first qualitative study to explore plain packaging of waterpipe tobacco products in a country that has existing waterpipe tobacco pictorial health warnings.
- We provide novel insights from both non-smokers and smokers into potential policy-relevant outcomes, particularly uptake and cessation of waterpipe tobacco smoking.
- Use of combined focus groups and in-depth interviews as qualitative methods offered rich understanding of perceptions related to waterpipe tobacco labelling, with respect to which contents pictorial health warnings might feature, and how design of waterpipe tobacco packs might be improved.
- Our sample of 90 individuals means we cannot generalize our findings, however we included a variety of participants, and achieved data saturation.
- While we explored projected rather than actual responses to existing and novel waterpipe tobacco pictorial health warnings with plain packaging, our findings could guide future experimental studies and assist policy-makers to improve waterpipe tobacco regulations.

KEY WORDS

Plain packaging, Health warnings, Waterpipe tobacco, Uptake, Cessation, Qualitative research

For peer review only - http://bmjopen.bmj.com/site/about/guidelines.xhtml

INTRODUCTION

The introduction of flavoured tobacco and the lack of regulatory policies have seen waterpipe tobacco smoking (WTS) increase globally.[1] Misperceptions that WTS is a safe alternative to cigarette smoking may also have contributed to rising waterpipe tobacco (WT) use,[2] even though WTS causes respiratory illnesses, cardiovascular diseases and adverse perinatal outcomes. [3] These factors have helped WTS become more socially acceptable, especially among youth[4,5] and women.[6,7]

WT use has extended beyond the East, where it has been present for decades, and is increasingly popular in the West, where WTS rates have reached 10% among some young adult populations in the United States and the UK.[8,9] The WHO Eastern Mediterranean Region (EMR) remains home to the highest WTS rates worldwide;[1,10] in some EMR countries, WTS has surpassed cigarette smoking in females and adolescents.[11,12] In Egypt, adolescent girls (3.4%)[13] and university students (12.2%)[14] report higher WTS rates than their older counterparts (0.3% in women[13]and 6.2% in men[15]), and rurally-located Egyptian males smoke WT more (7.5%) than men living in urban regions (4.9%).[15] This global surge in WTS makes examining the perceived effectiveness of existing WT control policies important to inform a much needed WT regulatory framework.[16-18]

Applying health warnings to tobacco products can cost-effectively increase public awareness of smoking's risks, increase the likelihood of quitting among smokers, and deter smoking initiation among non-smokers.[19] These outcomes are mediated by several measures of effectiveness that have been organized, based on behavioural theories, within conceptual frameworks of health

For peer review only - http://bmjopen.bmj.com/site/about/guidelines.xhtml

warning impact.[20-23] In line with this evidence, guidelines for implementing Article 11 of the WHO's Framework Convention on Tobacco Control (WHO FCTC) call for on-pack pictorial health warnings (PHWs), and recommend plain packaging and increasing warning size.[24] Egypt, a signatory country to the WHO FCTC, has applied generic PHWs to waterpipe tobacco packs (WTPs).[25] Since 2008, a set of four PHWs has appeared on the bottom half of the front and back of WTPs; these warnings carry the quitline number and rotate every two years.[26] However, WTPs still depict colourful fruits and flavours in brand imagery.[26]

Several observational[27] and experimental studies,[20] suggest plain packaging with larger PHWs[28] could more effectively reduce tobacco smoking through increasing warning salience, making the packaging and smoking less appealing, and reducing misperceptions about product harm,[29,30] especially in non-smokers or non-established smokers.[31]Yet, while this evidence is encouraging, these studies have focussed largely on cigarettes and we know little about how PHWs could reduce non-cigarette tobacco use, particularly WTS.[32]

To our knowledge, only a few studies have examined the impact of WT PHWs: two online surveys from Canada and the United States,[33,34] two qualitative studies from the UK[35] and the EMR,[36] and one recent Egyptian survey.[37] The two online surveys tested hypothetical warnings shown on computer screens rather than on WTPs and examined the effectiveness of text-only versus PHWs.[33,34] Both studies found that PHWs had a modest impact on established waterpipe smokers.[33,34] The UK qualitative study found that when warnings increased in size and packs became less branded, participants felt WTPs were less attractive and warnings were more impactful.[35] EMR study participants reported that PHWs improved respondents' knowledge

For peer review only - http://bmjopen.bmj.com/site/about/guidelines.xhtml

about WTS health hazards.[36] These studies are important but were confined to waterpipe smokers;[33-36] while it is important to examine how WT PHWs might pertain to both smokers and non-smokers. The Egyptian study reported that only half of 1048 waterpipe smoker and non-smoker participants thought that existing PHWs on WTPs were visible; they expressed varying views on the effectiveness of WT PHWs across several measures (such as salience, credibility, perceived harm, affective reactions).[37] However, this survey did not examine whether participants perceived existing WT PHWs effective in deterring uptake or quitting of WTS.

Given rising WTS rates in Egypt, in 2015 the Tobacco Control Unit in the Egyptian Ministry of Health proposed amending PHW regulations and introducing plain packaging. Specifically, it recommended increasing PHW size to 80% of the pack surface, and removing colours and flavour imagery from tobacco packs. Preliminary evidence on the potential effects of this approach was required. Qualitative methods are particularly useful in exploring understudied areas.[38,39] To inform WT labelling policy, we conducted this qualitative study to explore how participants perceived existing PHWs on WTPs, assessed how they interpreted plain packaging of WT featuring enhanced PHWs, and probed perceptions of how existing and novel sets would affect uptake or cessation of WTS.

METHODS

Design

The study comprised ten focus group discussions and ten in-depth interviews that took place in urban and semi-urban regions in Cairo, and a rural area in Menoufia governorate. We utilized both focus groups and in-depth interviews as complementary approaches; focus groups explored

For peer review only - http://bmjopen.bmj.com/site/about/guidelines.xhtml

participants' interactions and whether and how consensus views evolved while in-depth interviews allowed detailed probing and deeper understanding of participants' views.[38,39] Some sessions were conducted in WTS usage settings, such as cafes, where we observed social and cultural dynamics of WTS and assessed WT PHW's visibility to others.

Our conceptual framework drew on the theory of planned behavior,[40] as outlined in IARC Handbooks of Cancer Prevention, Tobacco Control, Methods for Evaluating Tobacco Control Policies 2008.[23] We explored policy-relevant outcomes with respect to the perceived potential effect on WTS uptake and cessation, and probed factors related to PHW content and WTP design, including salience, affective reactions, perceived harm and credibility. This study was approved by the Ethical Review Board of the Faculty of Medicine, Ain Shams University (FMASU R 10/2015). We used SRQR guidelines for reporting qualitative research.[41]

Sample

Our sample comprised men and women, 18 years of age or older, who lived in rural, urban, and semi-urban locations. We included self-identified waterpipe smokers (exclusive WT or dual users of WT and cigarettes) and non-smokers (non-users of any tobacco product), as we were interested in how warnings could influence WTS initiation as well as cessation. Participants were recruited using snowball sampling;[42] we explained the study purpose to people who made contact, invited them to participate in a one-on-one interview or focus group, and then set a meeting date and time. Participants did not discernibly differ by type of interview chosen.

In total, 90 individuals participated, including 80 in homogenous focus groups (with respect to

For peer review only - http://bmjopen.bmj.com/site/about/guidelines.xhtml

age, gender, smoking status) with 6–8 individuals per group, and 10 in in-depth interviews **(see Supplementary Table 1)**. As WT use in Egypt is generally higher among males,[15] more males participated in our sessions. **Supplementary Table 2** contains details of participants' demographic characteristics.

Tools

Interview Guide

We developed the interview guide in Egyptian colloquial Arabic and incorporated qualitative measures used to assess tobacco labelling policies.[23] We pilot tested the interview guide for clarity and comprehensiveness, tested the appropriateness of our prompts and questions after the pilot sessions, and made modifications following discussions with the research team. We used the same guide with focus groups and in-depth interviews and probed participants' experiences of WTS, their knowledge of WT PHWs, their views on the existing and novel PHWs on WTPs, and their perceptions of placing PHWs on waterpipe devices **(see Supplementary for interview guide).** In this article, we focus on discussing PHWs on WTPs.

Pictorial Health Warnings on Waterpipe Tobacco Packs

The existing PHWs depicted cancers of lung, throat, mouth, and face**,** covered 50% of the lower surface of the front and back of the WTP against a colourful background depicting fruit and flavour imagery. The warnings included pictures, generic text and the quitline number **(Figure 1A)**.

We adapted novel PHWs from a health warning database[43] and followed WHO FCTC

For peer review only - http://bmjopen.bmj.com/site/about/guidelines.xhtml

recommendations for plain packaging[24,44] building on the proposal of the Tobacco Control Unit of the Egyptian Ministry of Health; the PHW thus covered 80% of the upper surface of the front and back of the WTP against a dark uniform plain background not depicting any fruit or flavour imagery, with the remaining 20% depicting only the brand name in standardised font. The novel PHWs included pictures, text and the quitline number. We applied newly designed PHWs to used WTPs to promote authenticity. We pilot tested health warnings messages with corresponding images for clarity and comprehension: ("Smoking kills," "Smoking causes lung cancer", "Smoking causes clotting of blood vessels", "Smoking causes blindness", "Smoking during pregnancy harms fetus", and "Don't let your children inhale your smoke"). The warnings used were selected following discussions amongst the research team and feedback from pilot sessions, and reflected the best available evidence on WTS health outcomes.[45] WTS share common harms with those caused by cigarette smoking.[3] We sought topical content showing less severe harms than those depicted in existing PHWs. The four novel warnings selected included the effect of smoking during pregnancy on the fetus, effects of peripheral vascular diseases affecting the feet and eye, and effects on teeth and gums **(Figure 1 B)**. Although it was important to adapt the textual message to be waterpipe-specific, we did not test this in the study reported here; this was assessed separately in another study of our research project.

Data Collection

Data were collected from October 2015 to February 2016 at the Faculty of Medicine, Ain Shams University in Cairo (five focus groups and five in-depth interviews), and at participants' homes or in cafés for those in rural and semi-urban areas (five focus groups and five in-depth interviews). All participants received an information letter explaining the study and were asked

For peer review only - http://bmjopen.bmj.com/site/about/guidelines.xhtml

to provide verbal consent prior to each discussion or interview commencing. Participants were advised their data and identity would be confidential, and told they could withdraw from the study at any time.

Each focus group or interview was moderated by two of the co-authors (AM, WS, ME, and WM) and audio-recorded; each session was about one hour long. The facilitator and note taker regularly switched roles to promote reflection, and the wider team critically reflected on the interviews during team meetings. The facilitators followed standard procedures when discussing the interview guide topics and introduced the PHW stimuli when the relevant topic was opened for discussion. No further sessions were scheduled once data saturation had been reached, i.e. no new themes were being generated during the discussions.[46]

Analysis

Two authors independently transcribed verbatim the recorded sessions and compared the two transcripts to ensure inclusivity and accuracy (WH, HM); a third author (AM) resolved any discrepancies. Considering the identical aims and topics explored, data from focus groups and interviews were analyzed together. We analysed the data using a thematic approach.[47,48] We coded transcripts as the study progressed using a three-phase process that began by organizing ideas in relation to the research questions, then involved independent reviews of transcripts to identify preliminary themes and create an initial coding list.[49] We finally independently refined this list (AM, AA), added new codes where appropriate, and developed broader themes, that one author (HM) then reviewed across all cases. We resolved minor inconsistencies during discussion sessions and after extensive reviews of transcripts before we finalized the themes and subthemes **(see Supplementary Table 3)**. In this article, we focus on policy-relevant outcomes

For peer review only - http://bmjopen.bmj.com/site/about/guidelines.xhtml

relevant to PHWs on WTPs as described above in methods "design".

RESULTS

The 90 participants in focus groups and interviews comprised more males (72.2%) than females (27.8%) and participants' mean age was 33.4 ±11.6 **(see Supplementary Table 2)**. We identified the overall themes: warning label content and pack design features, and discuss these in relation to WTS uptake and cessation comparing throughout existing and novel sets. We report below results from both focus groups and individual interviews. We cite exemplar quotations below and provide a more detailed set of quotations in **Supplementary Table 4**, where we also indicate the gender, age group, smoking status, and location of participants.

Most participants were aware of warning labels on WTPs and reported seeing these when purchasing or preparing their tobacco. However, those using waterpipes in cafes or were less likely to see WTPs as WT was prepared out of their sight *"I was downtown and we saw smokers in cafes but shisha is always served ready…I never saw the packs" (Female non-smoker, >25y, semi-urban).*

Warning label content – perceived likely effect of existing and novel PHWs

Participants who were aware of existing WTP warnings recalled these as disturbing; however, several felt these warnings had limited impact. Many recalled the lung cancer PHW and the text "smoking is hazardous to health and causes death" as the most believable warning, yet also the least impactful, because of wearout: *"…the other warnings like the lung cancer one…people got used to them after a while" (Male smoker, >25y, rural).* Participants found the existing

For peer review only - http://bmjopen.bmj.com/site/about/guidelines.xhtml

warnings, which all featured cancer-related harms, frightening and disgusting, and non-smokers, particularly, avoided looking at them. Nonetheless, several questioned the harms existing PHWs featured and saw these as exaggerated: *"I don't want to look at it from near or far…they want to send us a message that it is harmful but in an awful and overstating way" (Female, smoker, <25y, urban).*

Some also found existing warnings difficult to understand: *"It looks like a bad thing but it is not clear what it is" (Female, non-smoker, >25y, semi-urban"*. These participants saw existing PHWs as unconvincing and exaggerated as they had not seen such conditions in real life and sometimes knew people who had smoked for many years with apparent impunity: *"I have seen the warning on the packs but I have never seen anything such as that in real life…I know a person who has been smoking since the eighties and nothing happened to him" (Male smoker, >25y, rural).*

Participants often denied risks associated with occasional smoking; one exempted himself from harm on the grounds that he did not smoke heavily and had seen no direct evidence of the harms presented in PHWs: *"I don't think I can be affected by smoking, because I don't smoke heavily; only once a day, besides...we have never seen the conditions in the warnings in real life" (Male smoker, >25y, rural).* Several thought harms would not occur until they were older, and believed that, if and when they experienced these, they could quit.

By contrast, most participants favoured the new PHWs, which they first viewed during the interview sessions. They found these clearer, more understandable and realistic than existing PHWs, and more likely to capture their attention. Participants also commented that the new

For peer review only - http://bmjopen.bmj.com/site/about/guidelines.xhtml

PHWs were easily understood even without text and thus likely to be effective among people with varying literacy: *"This one is more realistic for its purpose and understandable; even without any text…it is a more convincing warning" (Female non-smoker, >25y, semi-urban).*

Specifically, participants found the new warnings featuring more immediate, proximal risks had the strongest perceived impact, particularly those showing harmful effects on teeth. Female participants also felt strongly affected by the PHW illustrating harm to unborn babies, which forced them to confront the harm they imposed on others: *"The baby warning is effective because it is not about me anymore…This is something way more important than me…I fear for my kid more than I fear for myself" (Female non-smoker >25y, semi-urban).* However, some continued to see images showing potential harms they may experience still as overstated and unlikely to happen in the near future: *"This didn't happen before to anyone (foot warning)…we're still young…we won't get this" (Female smoker, <25y, urban).*

Although participants saw the new PHWs as more effective, they suggested improvements to the warning content. In line with earlier comments about perceived exaggeration, they sought greater credibility: *"It is very important that you convince me…put something there that I'll believe" (Male smoker, >25y, semi-urban).* They suggested presenting testimonials: "*Show smokers live people who were damaged because of shisha smoking and others who quit and improved*" (Male non-smoker, >25y, rural).

Participants recommended illustrating the effects WTS has on women by featuring relevant cancers and congenital diseases: "*Direct warnings addressing women like cancer of the breast or the uterus or congenital anomalies to the fetus" (Female, non-smoker, >25y, semi-urban"*. Some

For peer review only - http://bmjopen.bmj.com/site/about/guidelines.xhtml

also thought PHWs targeting women could encourage them to persuade their partners to quit.

Several participants thought PHWs should target youth before they start smoking and suggested warnings showing how WTS smokers' social relationships (e.g., sexual dysfunction warnings) would have high impact: *"We want a real effect that already happened…like for example the side effects on sexual functioning…that will definitely affect smokers" (Male non-smoker, >25 y, rural).*

In addition, participants also suggested printing text warnings including details of the hazardous ingredients of smoke, with more information on health risks and cessation options inserted on the inside or outside of the WTP, together with external PHWs would promote cessation behaviour, and would enhance the impact of novel warnings.

Design Features of WTPs – perceived likely effect of existing and novel PHWs

As well as responding to the different warning content, participants also noted that the different design elements used in the novel PHWs had improved the impact these had. Several commented on how bright colours and fruit imagery deflected attention away from PHWs and promoted experimentation: *"The peach drawing is appetizing…* [and] *drawing attention away from the warning…better put the warning on the top! The pack should be dark…this colour is very bright… just like bonbon packs" (Female non-smoker,>25y, semi-urban)*. They found it difficult to associate fruit flavours with harm and thought the images invited trial: *"The pictures and the smell of fruits make a passerby want to try them all"(Female non-smoker,>25y, semi-urban).*

By contrast, participants thought the plain background, contrasting colours, absence of fruit and

For peer review only - http://bmjopen.bmj.com/site/about/guidelines.xhtml

flavour images, and larger warning images shown on the proposed new PHWs increased impact, reduced distraction, and encouraged participants to look more closely at the pack. One noted: "*Here the picture is bigger and the text has a clear message…together with this dark colour…it makes me focus only on the warning…all this makes it more effective" (Male smoker, <25y, urban).* Together, the altered content and enhanced design attributes increased participants' perceptions of the impact the novel PHWs would have.

Overall, most waterpipe smoker and non-smoker participants thought PHWs would deter non-smokers from trying WTS but were less optimistic about the effects on smokers. One noted: "*If we (non-smokers) lusted to smoke it and saw pictures like these…we won't smoke, but those who do actually smoke already would be indifferent" (Male non-smoker, >25, urban).* Smokers themselves also felt PHWs had less effect because they had become accustomed to seeing the images: *"I used to think about the hazards a lot when the pictures first appeared…then I got used to them…I don't pay them attention anymore" (Male smoker, <25y, semi-urban).* Others reported using stickers to obscure PHWs or avoiding packs with PHWs they found particularly confronting: *"I avoid buying the picture of the tongue in particular* (referring to the mouth cancer warning)" *(Male smoker, <25y, semi-urban)*

As with the current PHWs, most waterpipe smoker and non-smoker participants thought the proposed PHWs would have a stronger effect on non-smokers than on long-term smokers, though some indicated they would avoid some warnings and may reduce their WTS: *"If I go to buy moassel (waterpipe tobacco) and found this pack, I'll go to another shop to buy another one…if I don't find a picture that makes me comfortable…I won't smoke that day…but if they're*

For peer review only - http://bmjopen.bmj.com/site/about/guidelines.xhtml

all like this…I guess I'll try to quit…or… at least decrease my habit" (Male smoker, >25 y, rural). In general, participants thought that the new PHW set had greater potential to deter WTS, especially among new smokers: *"If I'll smoke and saw it… for sure I won't smoke at the moment…it's disgusting" (Male non-smoker, <25y, rural).*

DISCUSSION

Our qualitative study found that participants privileged the short-term benefits they received over the longer-term risks they faced. They saw existing PHWs as less likely to influence long-term smokers, whose prolonged and repetitive exposure to PHWs appeared to have diminished their response to these aversive stimuli.[50] Older adult waterpipe users typically dismissed risks associated with WTS, especially if they had not experienced any health effects. This finding is consistent with other studies that found age is negatively associated with perceived risks of smoking and with attention to either graphic or text warnings on tobacco packages.[51]

However, the existing PHWs and the novel PHWs tested appeared more likely to influence non-smokers and less-experienced smokers by creating awareness of the health risks WTS presents. These findings are in line with previous research[31] and address McNeill et al.'s call in their recent review on plain packaging for further research into the effects of tobacco packaging on smoking uptake,[52] and we provide preliminary evidence that presenting WT in plain packaging could deter non-smokers from experimenting with WTS.

Our findings that novel warnings with clear and large visual elements could challenge perceived immediate benefits and enhance risk perception and acceptance is consistent with earlier work

For peer review only - http://bmjopen.bmj.com/site/about/guidelines.xhtml

and suggest presenting PHWs on WTS products featuring plain packaging could cue cessation.[35] More generally, our findings suggest a warning evaluation and regular refreshment programme may be important to ensure warning salience and avoid wearout.

Although existing PHWs induced strong negative emotional reactions, several participants viewed these warnings as exaggerated and felt the health risk depicted was unlikely to occur. While PHWs that arouse fear may increase risk perceptions, they did not necessarily promote greater message acceptance; our findings are thus consistent with earlier studies that reported highly confronting PHWs could have little effect on behaviour or even elicit adverse responses,[53,54] although other studies suggest that the higher negative affect aroused, the stronger the response elicited.[55] Our findings also show a complex relationship between the emotional response elicited and the salience and perceived impact of a message. The PHW depicting oral harm was minimally disturbing, yet participants saw it as the strongest and the most salient warning; by contrast, participants regarded the confronting vascular harm PHW as less effective because the condition was less salient and seen as more distal. As well as the image salience and affect aroused, the temporal framing is important and PHWs illustrating short-term harms were thought more effective. Our findings thus support earlier studies analysing the relationship between risk perception, believability, and temporal distance.[56]

Young adults and women found gender- and age-specific messages more persuasive. Evidence that WTS could be predicted by age and gender-related indicators,[57] suggests strategies targeting young adults and women are crucially important, particularly as these demographics perceive messages differently from older adults.[58] For example, messages about negative health effects

For peer review only - http://bmjopen.bmj.com/site/about/guidelines.xhtml

from passive smoking, sexual dysfunction and intimacy, miscarriages, and harmful effects on infants and children were considered more persuasive among younger adults and women than other messages.

Another plausible approach suggested by participants in our study was to develop a visible and sensible message in both text and graphic formats that target waterpipe smokers with detailed cessation information inserted on the WTP. This recommendation is consistent with that of an earlier study that found that combined text and graphic warnings elicit adverse reactions to smoking among non-smokers and smokers,[59] especially on the sustainability of quit behaviour.[60] In line with these suggestions, the design of novel warnings helped make the cessation quit line more prominent by the contrasting dark plain background.

We also found specific design elements that could inform future PHW development. Some participants thought plain packs increased the salience and effectiveness of the warnings, and reduced the appeal of the packaging and misperceptions of harm. These findings are consistent with the literature on plain packaging.[44,52] Evidence that flavoured cigarettes reduce harm perceptions appears relevant to WT[61,62] as participants noted that images of fruits or appealing flavours attracted them to WTS and deflected their attention from warning information. The novel warnings tested were presented as vivid images with a contrasting dark plain background and had no association with fruits or flavours. These PHWs had a stronger visual impact and were considered more salient than the PHWs currently used in Egypt. Some recommended that attractive imagery and flavour attributes should be banned. Furthermore, clarity of the pictures and presenting them in larger sizes played a role in enhancing acceptance and risk perception

For peer review only - http://bmjopen.bmj.com/site/about/guidelines.xhtml

among participants, a finding also reported by Jawad and colleagues.[35]

See **Strengths and Limitations** in **Supplementary file (page 8)**.

Conclusions

PHWs on WTPs have the potential to reduce uptake and cue quit attempts but might be more effective if PHWs used more impactful designs. Specifically, PHWs using contrasting background colours and plain packaging, offering no association to fruits or flavours, targeting age and gender, displaying proximal health risks, might enhance both warning impact and risk perception. Our exploratory study provides preliminary evidence of the potential effectiveness of WT products that carry enhanced warnings with plain packaging and suggests alternative designs and contents that could be further developed and tested in other studies. Policymakers designing and implementing health warnings on WT products now have clearer evidence on which to base their decisions.

For peer review only - http://bmjopen.bmj.com/site/about/guidelines.xhtml

ACKNOWLEDGEMENTS The authors are grateful to the participants of this research and whose responses provided the basis for this article. We thank Joe Petrik for editing and proofreading an earlier version of this manuscript. Both the current set of PHWs and the alternative set of PHWs presented in this study were used after signing a written partnership agreement between the Tobacco Control Unit in the Egyptian Ministry of Health and the research team.

AUTHOR CONTRIBUTORS AM conceived and designed the study and developed its tools. AM, ME, WS, WM carried out the study. SL and AM sought necessary approvals for using the existing set of PHWs. WM and HM transcribed the data. AA, HM, AM and JH analyzed the data. AM, HM, AA and JH drafted the paper. All authors critically revised contents and provided final approval on the submitted manuscript.

FUNDING This work was supported by the International Development Research Center (IDRC) through the American University of Beirut, the Tobacco Control Research Group, to study waterpipe tobacco smoking prevention and intervention programs in the region, as a part of the project "Shaping Research for Health in the Arab World: A Systems and Network Approach to Advance Knowledge, Inform Policy, and Promote Public Health" (Award 102518)

COMPETING INTERESTS None declared.

ETHICS APPROVAL This study was approved by the Research Ethics Committee of Faculty of Medicine, Ain Shams University (FMASU R 10/2015).

CHECKLIST FOR REPORTING GUIDELINES We used SRQR guidelines for reporting qualitative research.

For peer review only - http://bmjopen.bmj.com/site/about/guidelines.xhtml

DATA SHARING STATEMENT No additional data are available.

REFERENCES

1. Maziak W, Ben Taleb Z, Bahelah R, et al. The global epidemiology of waterpipe smoking. *Tob Control* 2015,24(1):i3-i12. doi:10.1136/tobaccocontrol-2014-051903.
2. Jawad M. Waterpipe Tobacco Smoking: A less Harmful Alternative? In: Loddenkemper R, Kreuter M (eds): The Tobacco Epidemic, ed 2, rev. and ext. Prog Respir Res. Basel, Karger, 2015,42:252-257. doi:10.1159/000369505.
3. Waziry R, Jawad M, Ballout RA, et al. The effects of waterpipe tobacco smoking on health outcomes: an updated systematic review and meta-analysis. *Int J Epidemiol* 2017;46(1):32-43. doi:10.1093/ije/dyw021.
4. Soule EK, Lipato T, Eissenberg T. Waterpipe tobacco-smoking: a new smoking epidemic among the young? *Curr Pulmonol Rep* 2015;4(4):163-172. doi:10.1007/s13665-015-0124-6.
5. Akl EA, Ward KD, Bteddini D, et al. The allure of the waterpipe: a narrative review of factors affecting the epidemic rise in waterpipe smoking among young persons globally. *Tob Control* 2015;24:i13–i21. doi:10.1136/tobaccocontrol-2014-051906.
6. Akl EA, Gunukula SK, Aleem S, et al. The prevalence of waterpipe tobacco smoking among the general and specific populations: a systematic review. *BMC Public Health* 2011;11:244. doi:10.1186/1471-2458-11-244.
7. Khalil J, Afifi R, Fouad FM, et al. Women and Waterpipe Tobacco Smoking in the Eastern Mediterranean Region: Allure or Offensiveness: *Women & Health* 2013;53(1):100-116. doi:10.1080/03630242.2012.753978.
8. Cobb C, Ward KD, Maziak W, et al. Waterpipe tobacco smoking: An emerging health crisis in the United States. *Am J Health Behav* 2010;34(3),275–285. PMCID: PMC3215592.

For peer review only - http://bmjopen.bmj.com/site/about/guidelines.xhtml

9. Grant A, Morrison R, Dockrell RJ. The Prevalence of waterpipe (Shisha, Narghille, Hookah) use among adults in great britain, and factors associated with waterpipe use: data from cross-sectional online surveys in 2012 and 2013. *Nicotine Tob Res* 2014;16(7): 931–938. doi:10.1093/ntr/ntu015.

10. Maziak W, Nakkash R, Bahelah R, et al. Tobacco in the Arab world: old and new epidemics amidst policy paralysis. *Health Policy Plan* 2013;29:784–94. doi.org/10.1136/tobaccocontrol-2014-051903.

11. Mostafa A, El Houssinie M, Aboul Foutouh A. Multiple Tobacco Use among Young Adult Waterpipe Smokers in Egypt. *East Mediterr Health J* 2018;24(x):xxx–xxx. https://doi.org/2018.24.2.XX. In press.

12. Jawad M, Lee JT, Millett C. Waterpipe Tobacco Smoking Prevalence and Correlates in 25 Eastern Mediterranean and Eastern European Countries: Cross-Sectional Analysis of the Global Youth Tobacco Survey. *Nicotine Tob Res* 2016;18(4):395-402. doi:10.1093/ntr/ntv101.

13. El Awa F, Fouad H, El Naga RA, et al. Prevalence of tobacco use among adult and adolescent females in Egypt. *East Mediterr Health J* 2013;19(8):749–54. PMID:24975361.

14. Shisha and smokeless tobacco use among university students in Egypt: prevalence, determinants, and economic aspect. A joint report by the Egyptian Ministry of Health and Population and the World Health Organization 2014. http://applications.emro.who.int/dsaf/EMROPUB_2014_EN_1752.pdf?ua=1&ua=1 (accessed May 2017).

15. World Health Organization: Global Adult Tobacco Survey: Egypt Country Report 2009. http://www.who.int/tobacco/surveillance/gats_rep_egypt.pdf (accessed September 2017).

For peer review only - http://bmjopen.bmj.com/site/about/guidelines.xhtml

16. World Health Organization Framework Convention on Tobacco Control: Control and prevention of waterpipe tobacco products (document FCTC/COP/7/10). In: Conference of the parties to the WHO framework convention on tobacco control, seventh session, Delhi, India, 7-12 November 2016. World Health Organization, Geneva. http://www.who.int/fctc/cop/cop7/FCTC_COP_7_10_EN.pdf (accessed May 2017).

17. Jawad M, El Kadi L, Mughrabil S, et al. Waterpipe tobacco smoking legislation and policy enactment: a global analysis. *Tob Control* 2015;24(1):i60-i65. doi:10.1136/tobaccocontrol-2014-051911.

18. Salloum RG, Asfar T, Maziak W. Toward a Regulatory Framework for the Waterpipe. Am *J Public Health* 2016;106(10):1773-7. doi:10.2105/AJPH.2016.303322.

19. Hammond D. Health warning messages on tobacco products: a review. *Tob Control* 2011;20(5):327–337. doi.org/10.1136/tc.2010.037630.

20. Noar SM, Hall MG, Francis DB, et al. Pictorial cigarette pack warnings: a meta-analysis of experimental studies. *Tob Control* 2016;25(3):341-54. doi:10.1136/tobaccocontrol-2014-051978.

21. Yong HH, Borland R, Thrasher JF, et al. Mediational pathways of the impact of cigarette warning labels on quit attempts. *Health Psychol* 2014;33(11):1410–20. doi: 10.1037/hea0000056.

22. Emery LF, Romer D, Sheerin KM, Jamieson KH, Peters E. Affective and Cognitive Mediators of the Impact of Cigarette Warning Labels. *Nicotine Tob Res* 2014;16(3):263–9. doi.org/10.1093/ntr/ntt124.

For peer review only - http://bmjopen.bmj.com/site/about/guidelines.xhtml

23. IARC Handbooks of Cancer Prevention, Tobacco Control. Methods for Evaluating Tobacco Control Policies 2008; 12. Lyon, France. http://www.iarc.fr/en/publications/pdfs-online/prev/handbook12/Tobacco_vol12.pdf (accessed April, 2015).

24. World Health Organization. Guidelines for implementation of Article 11 of the WHO Framework Convention on Tobacco Control (Packaging and labelling of tobacco products) 2008. http://www.who.int/fctc/guidelines/article_11.pdf?ua=1 (accessed April, 2015).

25. Mostafa A, Kashiwabara M. Tobacco packaging and labelling policies in countries of the Eastern Mediterranean and Western Pacific Regions: Post-deadline assessment of the time-bound measures of WHO FCTC Article 11. *Tobacco Prevention & Cessation* 2016;2(November):78. doi:10.18332/tpc/66793.

26. World Health Organization: Tobacco control country profiles 2017. Egypt. http://www.who.int/tobacco/surveillance/policy/country_profile/egy.pdf?ua=1 (accessed 15 December 2017).

27. Stead M, Moodie C, Angus K, et al. Is Consumer Response to Plain/Standardised Tobacco Packaging Consistent with Framework Convention on Tobacco Control Guidelines? A Systematic Review of Quantitative Studies. *PLoS ONE* 2013;8(10): e75919. doi:10.1371/journal.pone.0075919.

28. Wakefield M, Coomber K, Zacher M, et al. Australian adult smokers' responses to plain packaging with larger graphic health warnings 1 year after implementation: results from a national cross-sectional tracking survey. *Tob Control* 2015;24(2):ii17–ii25. doi:10.1136/tobaccocontrol-2014-052050.

29. Durkin S, Brennan E, Coomber K, et al. Short-term changes in quitting-related cognitions and behaviours after the implementation of plain packaging with larger health warnings:

For peer review only - http://bmjopen.bmj.com/site/about/guidelines.xhtml

findings from a national cohort study with Australian adult smokers. *Tob Control* 2015;24:ii26-ii32. doi:10.1136/tobaccocontrol-2014-052058.

30. Mays D, Niaura RS, Evans WD, et al. Cigarette packaging and health warnings: the impact of plain packaging and message framing on young smokers. *Tob Control* 2015;24(e1):e87-92. doi: 10.1136/tobaccocontrol-2013-051234.

31. Munafo MR, Roberts N, Bauld L, et al.: Plain packaging increases visual attention to health warnings on cigarette packs in non-smokers and weekly smokers but not daily smokers. *Addiction* 2011;106(8):1505-10. doi:10.1111/j.1360-0443.2011.03430.x

32. Ross JC, Noar SM, Sutfin EL: Systematic Review of Health Communication for Non-Cigarette Tobacco Products. *Health Communication* 2017; doi:10.1080/10410236.2017.1407274.

33. Mohammed H T. The efficacy of viewing health warnings on shisha smoking among shisha smokers. https://uwspace.uwaterloo.ca/bitstream/handle/10012/7419/Mohammed_HebaTallah.pdf?sequence=1 (accessed September, 2017).

34. Islam F, Salloum RG, Nakkash R, et al.: Effectiveness of health warnings for waterpipe tobacco smoking among college students. *Int J Public Health* 2016;61(6):709-715. doi: 10.1007/s00038-016-0805-0.

35. Jawad M, Bakir A, Ali M, et al.: Impact of Waterpipe Tobacco Pack Health Warnings on Waterpipe Smoking Attitudes: A Qualitative Analysis among Regular Users in London. *BioMed Res Int* 2015:745865.doi: 10.1155/2015/745865.

36. Salloum RG, Abu-Rmeileh N, Hamadeh R, et al.: Policy-Relevant Context of Waterpipe Tobacco Smoking among University Students in Six Countries Across the Eastern

For peer review only - http://bmjopen.bmj.com/site/about/guidelines.xhtml

Mediterranean Region: A Qualitative Study. *Asian Pac J Cancer Prev* 2017;18(9): 2533-2540. doi:10.22034/APJCP.2017.18.9.2533.

37. Mostafa A, Mohammed HT. Graphic health warnings and their best position on waterpipes: A cross-sectional survey of expert and public opinion. *Tobacco Prevention & Cessation* 2017; 3(June):116. doi: 10.18332/tpc/70873.
38. Jones R: Why do qualitative research? *BMJ* 1995; 311(6996):2. PMCID: PMC2550075.
39. Pope K, Mays R: Qualitative research: Reaching the parts other methods cannot reach: An introduction to qualitative methods in health and health services research. *BMJ* 1995; 311 (6996):42-5. PMCID: PMC2550091.
40. Ajzen I. The theory of planned behavior. Organ Behav Hum Decis Process 1991;50(2):179–211. https://doi.org/10.1016/0749-5978(91)90020-T.
41. O'Brien BC, Harris IB, Beckman TJ, et al. Standards for reporting qualitative research: a synthesis of recommendations. *Acad Med* 2014;89(9):1245-1251.
42. Feldman MS, Bell J, Berger MT. Gaining Access: A Practical and Theoretical Guide for Qualitative Researchers, Altamira Press 2003, Oxford, UK.
43. World Health Organization Framework Convention on Tobacco Control. Health Warnings Database: http://www.who.int/tobacco/healthwarningsdatabase/en/ (accessed April 2015).
44. World Health Organization. Evidence, Design and Implementation of Plain Packaging at: http://apps.who.int/iris/bitstream/10665/207478/1/9789241565226_eng.pdf?ua=1 (accessed December 2016).
45. Akl EA, Gaddam S, Gunukula SK, et al. The effects of waterpipe tobacco smoking on health outcomes: A systematic review. *Int J Epidemiol* 2010;39:834–857. doi:10.1093/ije/ dyq002.

For peer review only - http://bmjopen.bmj.com/site/about/guidelines.xhtml

46. Walker JL. Research column. The use of saturation in qualitative research. *Can J Cardiovasc Nurs* 2012;22:37-46. PMID: 22803288.

47. Braun V, Clarke V. Using thematic analysis in psychology. *Qualitative Research in Psychology 2006;* 3(2,): 77–101. doi:10.1191/1478088706qp063oa.

48. Pope C, Ziebland S, Mays N: Qualitative research in health care: Analysing qualitative data. *BMJ* 2000;320(7227):114–116. PMCID: PMC1117368

49. Boyatzis RE: Transforming qualitative information: thematic analysis and code development California: Sage Publications; 1998.

50. White V, Bariola E, Faulkner A, et al. Graphic Health Warnings on Cigarette Packs: How Long Before the Effects on Adolescents Wear Out? *Nicotine Tob Res* 2015;17(7):776-83. doi:10.1093/ntr/ntu184.

51. Robert ME, Peters E, Ferketich AK, et al. The Age-related Positivity Effect and Tobacco Warning Labels. *Tob Regul Sci* 2016;2(2):176-185. doi:10.18001/TRS.2.2.8.

52. McNeill A, Gravely S, Hitchman SC, et al. Tobacco packaging design for reducing tobacco use. *Cochrane Database of Systematic Reviews* 2017, Issue 4. Art. No.: CD011244. doi:10.1002/14651858.CD011244.pub2.

53. Peters GJ, Ruiter RA, Kok G. Threatening communication: A critical re-analysis and a revised meta-analytic test of fear appeal theory. *Health Psychology Review* 2013;7(1):S8–S31. doi:10.1080/17437199.2012.703527.

54. Thrasher J, Swayampakala K, Borland R, et al. Influences of Self-Efficacy, Response Efficacy, and Reactance on Responses to Cigarette Health Warnings: A Longitudinal Study of Adult Smokers in Australia and Canada. *Health Commun* 2016;31(12):1517-1526. doi:10.1080/10410236.2015.1089456.

For peer review only - http://bmjopen.bmj.com/site/about/guidelines.xhtml

55. Droulers O, Gallopel-Morvan K, Lacoste-Badie S, et al. The influence of threatening visual warnings on tobacco packaging: Measuring the impact of threat level, image size, and type of pack through psychophysiological and self-report methods. *PLoS One* 2017;12(9):e0184415. doi:10.1371/journal.pone.0184415.

56. Hoek J, Hoek-Sims A, Gendall P. A qualitative exploration of young adult smokers' responses to novel tobacco warnings. *BMC Public Health* 2013;13:609. doi: 10.1186/1471-2458-13-609.

57. Jaber R, Madhivanan P, Khader Y, et al. Predictors of waterpipe smoking progression among youth in Irbid, Jordan: A longitudinal study (2008-2011). *Drug Alcohol Depend* 2015;153:265-70. doi:10.1016/j.drugalcdep.2015.05.008.

58. Koval J, Aubut J, Pederson L, et al. The Potential Effectiveness of Warning Labels on Cigarette Packages. The Perceptions of Young Adult Canadians. *Can J Public Health* 2005; 96(5):354. doi:10.1016/j.drugalcdep.2015.05.008.

59. Singh A, Owusu-Dabo E, Britton J, et al. "Pictures don't lie, seeing is believing": exploring attitudes to the introduction of pictorial warnings on cigarette packs in Ghana. *Nicotine Tob Res* 2014;16(12):1613-9. doi:10.1093/ntr/ntu127.

60. Thrasher J, Swayampakala K, Cummings M, et al. Cigarette package inserts can promote efficacy beliefs and sustained smoking cessation attempts: A longitudinal assessment of an innovative policy in Canada. *Preventive Medicine* 2016; 88:59–65. doi:10.1016/j.ypmed.2016.03.006.

61. Rising J, Alexander L. Marketing of menthol cigarettes and consumer perceptions. *Tob Induc Dis* 2011;9(1): S2. doi:10.1186/1617-9625-9-S1-S2.

62. Hammond D, Parkinson C. The impact of cigarette package design on perceptions of risk.

For peer review only - http://bmjopen.bmj.com/site/about/guidelines.xhtml

Journal of Public Health 2009;31(3):345-53. doi:10.1093/pubmed/fdp066.

For peer review only

For peer review only - http://bmjopen.bmj.com/site/about/guidelines.xhtml

Figure 1. Pictorial Health Warnings used in this study

For peer review only

For peer review only - http://bmjopen.bmj.com/site/about/guidelines.xhtml

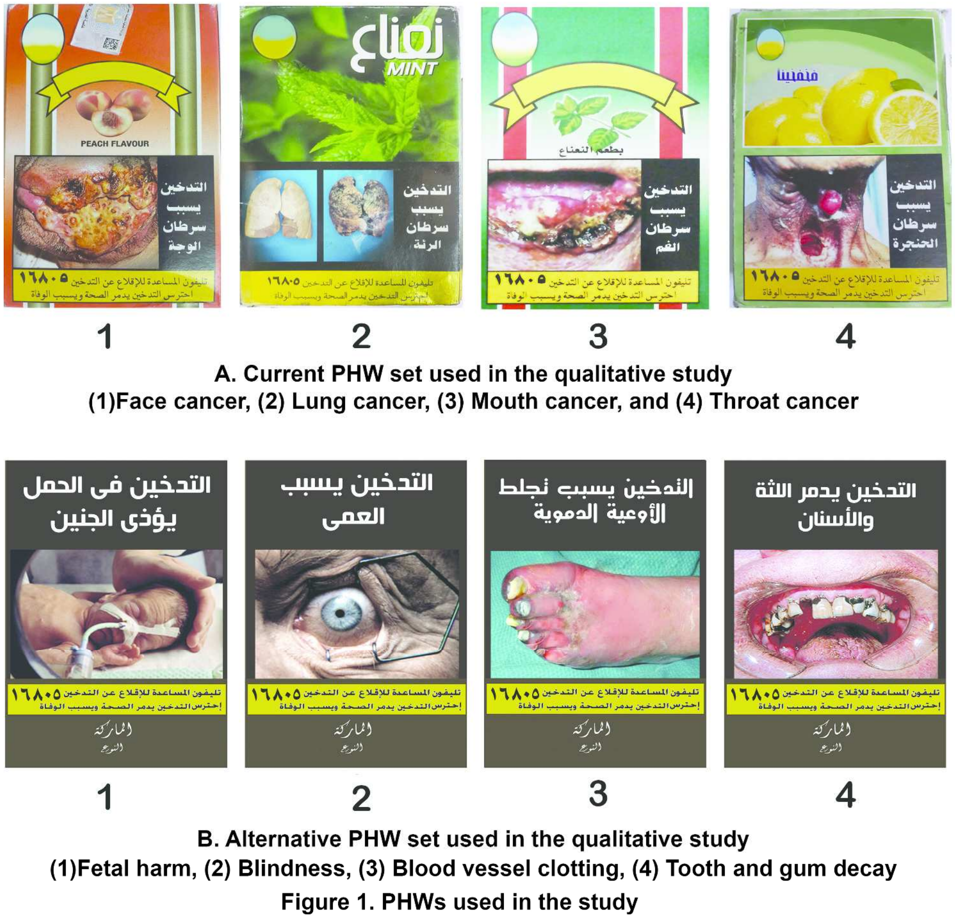

Figure 1. Pictorial Health Warnings used in this study

297x300mm (300 x 300 DPI)

Supplementary

Table 1. Total number of interviews and focus group discussions conducted by demographic profile of participants

	FGDs (10, total participants=80)				IDIs (10)			
	Males (7)		Females (3)		Males (7)		Females (3)	
Age	S	NS	S	NS	S	NS	S	NS
	(5)	(2)	(2)	(1)	(6)	(1)	(2)	(1)
18-24	2	1	1		3	1	1	
25+	3	1	1	1	3		1	1

FGDs=Focus group discussions, IDIs=In-depth interviews, S=Waterpipe smoker, NS=Non-smoker

For peer review only

Table 2. Sociodemographic characteristics and waterpipe smoking status of participants

	N=90	%
**Gender**		
Male	**65**	**72.2**
Female	**25**	**27.8**
**Age (range 18-73)**		
**Mean age ± standard deviation** 33.4 ± 11.6		
**Age group, years**		
18-24	**21**	**23.3**
25+	**69**	**76.7**
**Education level**		
Primary or lower	**16**	**17.7**
Secondary	**32**	**35.6**
Tertiary	**42**	**46.7**
**Occupation**		
Employed professional	**42**	**46.7**
Employed non-professional	**34**	**37.8**
In further study or seeking employment	**14**	**15.5**
**Residence**		
Rural	**20**	**22.2**
Semi-urban	**51**	**56.7**
Urban	**19**	**21.2**
**Smoking status**		
Waterpipe smoker	**62**	**68.9**
Non-smoker	**28**	**31.1**
**Ever noticed PHWs on WTPs**		
Yes	**53**	**58.9**
**Where**		
At cafés	**17**	**32.1**
At home	**11**	**20.8**
Buying tobacco	**25**	**47.2**

For peer review only - http://bmjopen.bmj.com/site/about/guidelines.xhtml

Interview guide *

**Interview guide questions**	**Parts relevant to this article**
**Introductory questions**<br>*In general, what do you think of waterpipe smoking?*<br>**Personal experiences of waterpipe smoking**<br>*Tell me about your waterpipe smoking, habits, duration, etc…Tell us more on how did you start smoking?*<br>*Could you describe the setting in which you smoked waterpipe last time?*	
**Awareness of pictorial health warnings**<br>*Have you ever noticed the pictorial health warnings on waterpipe tobacco packs?*<br>*Where did you see them? Can you tell us more about the setting?*<br>*How do they look like?*	√
**Positive and negative characteristics of the current/alternative set of PHW**<br>*Have you ever come across the warning on this pack before? What is your first impression about it?*<br>*What do you think/feel when you see it?*<br>*In your opinion, what is good and is bad about it? Why do you think so?*<br>*Do you think it is/would be effective? Why and how do you think so?*	√
**Reactions to placing PHW on the waterpipe device**<br>*What is your first impression about putting the warning in this spot?*<br>*What do you think/feel when you see there?*<br>*In your opinion, what is good and is bad about this spot? Why do you think so?*<br>*Do you think people will notice the PHW on this spot?*<br>*Would it be effective? Why and how do you think so?*	

***This guide contains core questions. Probing questions were used when necessary (examples include questions on salience, affective reactions, perceived harm, credibility)**

For peer review only - http://bmjopen.bmj.com/site/about/guidelines.xhtml

Table 3. Themes and subthemes identified from focus groups and in-depth interviews*

**Themes**	**Subthemes**	**Themes covered in this article**
General perceptions of waterpipe smoking	Factors contributing to WTS<br>Factors affecting WTS initiation and continuation<br>WTS setting<br>Patterns of WTS consumption<br>Social and cultural context of WTS<br>WTS compared to cigarette smoking<br>Tobacco product characteristics<br>Perceived health consequences<br>Reasons for quitting	
Views on pictorial health warning labels on waterpipe tobacco packs	General knowledge /awareness of PHWs on WTPs<br>Sources of awareness<br>Most salient/recalled labels, reasons<br>Cessation helpline<br>Perception of PHWs on WTPs motivating and discouraging factors related to viewing PHW labels	Partly
Perceptions of existing waterpipe tobacco warnings	General impressions, attitude (appeal, affective reactions, avoidance, wear out)<br>Positive and negative characteristics of design and content<br>Participants views of the most effective one in general<br>Possible effects on quitting and/or initiating WTS	√
Insights into novel waterpipe tobacco warnings	General impressions, attitude (appeal, affective reactions, avoidance)<br>Positive and negative characteristics of design and content<br>Participants views of the most effective one in general<br>Possible effects on quitting and/or initiating WTS	√
Avenues for development	Participant suggestions of improved label characteristics regarding content, design, location<br>Participant suggestions for other placement locations	Partly
Best location for inserting labels on the waterpipe device and accessories	Views behind choosing each location<br>Concerns about each location<br>Possible effects on quitting and/or initiating WTS	
Participant suggestions for reducing tobacco use in general and WTS in particular	Further strategies for improved regulations<br>Enforcing existing laws<br>Posting warnings in public and other places<br>Associate warnings with mass-media anti-tobacco campaigns, increase taxation and other suggestions	

*** Themes presented in this article were synthesized in respect to warning content and pack design and their perceived likely effect on uptake or cessation of waterpipe smoking, comparing existing versus novel warnings.**

For peer review only - http://bmjopen.bmj.com/site/about/guidelines.xhtml

Table 4. Selected participants' quotes representing views of the current and alternative pictorial health warning labels on waterpipe packs. Quotes are presented by gender, age group*, smoking status, and region of participants

Warning label content – perceived likely effect of existing and novel PHWs	
"The picture of a bent cigarette (i.e. representing sexual dysfunction) caught people's attention considerably…but the other warning…people got used to them after a while"	*(Male smoker, >25y, rural)*
"The most commonly known warning is that of the lungs"	*(Male smoker,>25y, semi-urban)*
"The picture is disgusting and frightening…"	*(Male non-smoker, <25y, rural)*
"Pictures are scary and unpleasant"	*(Male smoker, >25y, semi-urban)*
"The pictures make me sick…"	*(Male smoker, >25y, urban)*
"The warning shouldn't be that it causes cancer and that's it…that is just routine talk"	*(Male non-smoker, >25y, urban)*
"I just don't believe it…nobody reaches this stage"	*(Female smoker, <25y, urban)*
"I don't believe the warnings…I know smoking is harmful but not to that extent. This is unrealistic"	*(Male smoker, <25y, rural)*
"We want to see the normal lung beside the diseased one…but this one straight is not clear"	*(Male non-smoker, >25y, urban)*
"The pictures are not realistic, we never see such things in real life"	*(Male non-smoker, >25y, semi-urban)*
"The pictures are extremely exaggerated"	*(Male smoker, <25y, urban)*
"Is this a burnt piece of pizza? (face cancer warning)…this one won't be clear to illiterates…I didn't get it before reading the text beneath"	*(Male non-smoker, >25y, urban)*
"The picture is frightening and is very disgusting"	*(Male smoker, >25y, urban)*
"It can make me want to look closely to see what the illness shown is"	*(Female non-smoker, >25y, semi-urban)*
"For me, the most effective one is picture of teeth affection…it's very scary and upsetting. I see that content of the warning is much more important than size"	*(Male smoker, >25y, semi-urban)*
"With this shape (deformed teeth and gums) I may disturb my son when I come close to kiss him...now that will hurt me"	*(Male smoker, >25y, semi-urban)*
"The baby's picture may affect women more than me"	*(Male smoker, >25y, semi-urban)*
"If a pregnant woman saw this picture…she would fear for her unborn child to have anomalies"	*(Female smoker, >25y, semi-urban)*
"Explain the components of tobacco as glycerin and the hazards of each of them…tell them: try to smoke each component by itself and see what happens to you"	*(Male non-smoker, >25y, semi-urban)*
"Simplify the matter of quitting to people…especially those who have been smoking for a long time…"	*(Male nonsmoker, >25 y, rural)*
"If you talk to a smoker about their health…they wouldn't pay attention but there are other aspects as hazards to their children"	*(Male non-smoker,>25y, semi-urban)*
"The most effective thing that makes a smoker quit is that they themselves get ill or they see someone get ill"	*(Male smoker, >25y, semi-urban)*
" I saw on the internet a pack of cigarettes with a picture of a mother and her child, and on opening it the mother is separated from him. This a new effective idea."	*(Female smoker, <25 y, urban)*

For peer review only - http://bmjopen.bmj.com/site/about/guidelines.xhtml

“A pregnant woman or a mother may ask their smoker husband to stop”	*(Female non-smoker, >25y, semi-urban)*
"The only thing that can force me to stop smoking, even if I'm a heavy smoker is the baby affection"	*(Female smoker, <25 y, urban)*
“Warnings should target young people under 17 before they attempt smoking”	*(Male non-smoker, >25y, semi-urban)*
"Warnings concerning sexual dysfunction can affect young smokers”	*(Male non-smoker,>25y, semi-urban)*
”Focus on the damage that the family suffers…there are problems that can occur with the wife because of smoking…so smoking may endanger the family bonds”	*(Male smoker, >25y, semi-urban)*
“Pictures should be more realistic, and not only focus on severe side effects like cancers… like if warnings demonstrate just a fingernail affected due to smoking not all the hand, people will be more tempted to believe it"	*(Male smoker, <25 year, rural)*
Design Features of WTPs – perceived likely effect of existing and novel PHWs	
“The apples got my attention”	*(Female smoker, >25y, semi-urban)*
“After seeing this…(pointing to the fruit depicted on the pack)…it will be hard for me to link the idea with the damage in this picture”	*(Male non-smoker, <25y, rural)*
"Pictures of fruits are attractive and appetizing”	*(Male smoker, >25y, semi-urban)*
“If there is no flavored tobacco, I will quit"	*(Male smoker, <25y, urban)*
“I can’t see any flavors on it…I want to hold it closely to know what did they put there instead…if shisha is served as it is…without any flavors?!...I’ll quit”	*(Male smoker, <25y, urban)*
"If flavors were banned may be some will quit but warnings have no effect”	*(Male smoker, <25y, urban)*
"The pictures of fruits must be removed as they are appetizing"	*(Male non-smoker, <2, rural)*
"The dark background makes the writings clearer”	*(Male non-smoker,>25y, semi-urban)*
"The alternative pack is clearer regarding the color, the picture and the way of writing"	*(Male non-smoker, < 25 y, rural)*
"The black color makes the pack a lot chic"	*(Male smoker, <25y, urban)*
"The color of the package is dark and it conveys a sense of pessimism"	*(Male smoker, >25y, rural)*
"The bigger the picture, the better”	*(Male smoker, <25y, semi-urban)*
"The warning should be put on top because the pictures of fruits are drawing attention away from the warning”	*(Female non-smoker, >25y, semi-urban)*
"Oh! I didn’t realize there was a quit line on the pack before…this dark background made some kind of contrast… the text now is way clearer for me to read”	*(Female smoker<25y, urban)*
“I used to think about the hazards a lot when the pictures first appeared…then I got used to them…I don’t pay them attention anymore”	*(Male smoker, <25y, semi-urban)*
"I have seen the warnings too often that they no longer frighten me”	*(Male smoker, <25y, rural)*
"The pictures are horrible but people got used to them, if a nonsmoker wished to try and saw the warnings they may change their mind but for those who are already smokers, the warnings make no difference	” *(Male non-smoker,>25y, semi-urban)*
“Warnings influence the non-smokers much more than smokers, as non-smokers usually find the picture more disgusting”	*(Female, non-smoker, >25y, semi-urban)*

For peer review only - http://bmjopen.bmj.com/site/about/guidelines.xhtml

"I avoid buying the picture of the tongue in particular (referring to the mouth cancer warning)"	*(Male smoker, <25y, semi-urban)*
"People can go around all day looking for a certain picture (the lungs)"	*(Male smoker, >25y, rural)*
"Vendors used to sell the pack with free stickers to cover the warnings"	*(Male smoker,>25y, semi-urban)*
"No one would buy a pack with such warning (means warning picturing effect of smoking on children)"	*(Female smoker, >25y, semi-urban)*
"If the warning on the pack looks bad, we tend to throw the external package away and put tobacco in another package or may be order a certain warning"	*(Male smoker, >25y, rural)*
"The alternative pack may make some smokers reduce smoking"	*(Male non-smoker, <25y, rural)*
"A beginner can be affected compared to a person who has been smoking for 15 years and nothing happened to them as a result of smoking"	*(Male non-smoker, <25y, rural)*
"Non-smokers will be more affected by it, smokers won't be much affected"	*(Female, non-smoker, >25y, semi-urban)*
"It could affect those who want to start smoking, but the older smokers won't be affected much"	*(Male, smoker, <25y, semi-urban)*

***The age group of participants was categorized as > or < 25 years, because we were interested in highlighting the views of young adults who may be at higher risk of experiencing WTS or maybe non-established WT users yet, versus older adults who might more likely have established a smoking or non-smoking behaviour.**

For peer review only

Discussion-Strengths and Limitations

To our knowledge, this is the first qualitative study to assess smokers' and non-smokers' awareness and acceptance of currently used and novel WT PHWs with plain packaging. Our novel warnings simulated how PHWs appear (or could appear) on packs and devices, we explored projected rather than real-life responses. Experimental work could estimate the likely impact of our new label designs, including more targeted PHWs, on waterpipe smokers' risk perceptions, attitudes and likely cessation responses. Future research could also develop a more comprehensive analysis of factors motivating and reinforcing WTS uptake. Given our findings that PHWs have the potential to reduce WTS initiation, it is also important to test whether the PHW themes we developed could be used in wider health promotion campaigns to reduce the appeal and perceived acceptability of WTS. Future research could explore how WT warnings featuring waterpipe-specific messages would affect awareness and perceptions of WTS.

Our small sample means we cannot generalize our findings, though we note a sample of 90 individuals is still substantial, and saturation had been reached in the responses received. Despite these limitations, our study provides novel insights into the factors supporting WTS uptake, suggests themes PHWs could feature, and outlines how PHWs' format could be improved. We recognize this work programme faces challenges, given the limited resources and capacity in low- and middle-income countries; nonetheless, our findings represent an important step in supporting a comprehensive regulatory framework that reduces WTS and the harm this form of tobacco use causes.

For peer review only - http://bmjopen.bmj.com/site/about/guidelines.xhtml

Reporting checklist for qualitative study.

Based on the SRQR guidelines.

Instructions to authors

Complete this checklist by entering the page numbers from your manuscript where readers will find each of the items listed below.

Your article may not currently address all the items on the checklist. Please modify your text to include the missing information. If you are certain that an item does not apply, please write "n/a" and provide a short explanation.

Upload your completed checklist as an extra file when you submit to a journal.

In your methods section, say that you used the SRQR reporting guidelines, and cite them as:

O'Brien BC, Harris IB, Beckman TJ, Reed DA, Cook DA. Standards for reporting qualitative research: a synthesis of recommendations. Acad Med. 2014;89(9):1245-1251.

		Reporting Item	Page Number
	#1	Concise description of the nature and topic of the study identifying the study as qualitative or indicating the approach (e.g. ethnography, grounded theory) or data collection methods (e.g. interview, focus group) is recommended	1,3, 8 & 9
	#2	Summary of the key elements of the study using the abstract format of the intended publication; typically includes background, purpose, methods, results and conclusions	3, 4
Problem formulation	#3	Description and signifcance of the problem / phenomenon studied: review of relevant theory and empirical work; problem statement	3, 5-9
Purpose or research question	#4	Purpose of the study and specific objectives or questions	3, 8
Qualitative approach	#5	Qualitative approach (e.g. ethnography,	8, 9

For peer review only

and research paradigm		grounded theory, case study, phenomenolgy, narrative research) and guiding theory if appropriate; identifying the research paradigm (e.g. postpositivist, constructivist / interpretivist) is also recommended; rationale. The rationale should briefly discuss the justification for choosing that theory, approach, method or technique rather than other options available; the assumptions and limitations implicit in those choices and how those choices influence study conclusions and transferability. As appropriate the rationale for several items might be discussed together.	See 5 and Supplementary file page 8 for more on limitations
Researcher characteristics and reflexivity	#6	Researchers' characteristics that may influence the research, including personal attributes, qualifications / experience, relationship with participants, assumptions and / or presuppositions; potential or actual interaction between researchers' characteristics and the research questions, approach, methods, results and / or transferability	11, 12 for reflexivity<br>Researchers are trained or experienced in qualitative methods. Word count limited detailing qualifications and other items listed here.
Context	#7	Setting / site and salient contextual factors; rationale	8, 9, 11
Sampling strategy	#8	How and why research participants, documents, or events were selected; criteria for deciding when no further sampling was necessary (e.g. sampling saturation); rationale	9, 10, 12
Ethical issues pertaining to human subjects	#9	Documentation of approval by an appropriate ethics review board and participant consent, or explanation for lack thereof; other confidentiality and data security issues	9, 11
Data collection	#10	Types of data collected; details of data	10, 11

For peer review only

methods		collection procedures including (as appropriate) start and stop dates of data collection and analysis, iterative process, triangulation of sources / methods, and modification of procedures in response to evolving study findings; rationale	
Data collection instruments and technologies	#11	Description of instruments (e.g. interview guides, questionnaires) and devices (e.g. audio recorders) used for data collection; if / how the instruments(s) changed over the course of the study	10, 11
Units of study	#12	Number and relevant characteristics of participants, documents, or events included in the study; level of participation (could be reported in results)	9, 10
Data processing	#13	Methods for processing data prior to and during analysis, including transcription, data entry, data management and security, verification of data integrity, data coding, and anonymisation / deidentification of excerpts	11
Data analysis	#14	Process by which inferences, themes, etc. were identified and developed, including the researchers involved in data analysis; usually references a specific paradigm or approach; rationale	11, 12
Techniques to enhance trustworthiness	#15	Techniques to enhance trustworthiness and credibility of data analysis (e.g. member checking, audit trail, triangulation); rationale	12
Syntheses and interpretation	#16	Main findings (e.g. interpretations, inferences, and themes); might include development of a theory or model, or integration with prior research or theory	12<br>See 9 for theory
Links to empirical data	#17	Evidence (e.g. quotes, field notes, text excerpts, photographs) to substantiate	13-17

For peer review only

		analytic findings	
Intergration with prior work, implications, transferability and contribution(s) to the field	#18	Short summary of main findings; explanation of how findings and conclusions connect to, support, elaborate on, or challenge conclusions of earlier scholarship; discussion of scope of application / generalizability; identification of unique contributions(s) to scholarship in a discipline or field	18-21<br>See also 3, 5 and Supplementary file page 8 for more on limitations
Limitations	#19	Trustworthiness and limitations of findings	5 and Supplementary file page 8 for more on limitations
Conflicts of interest	#20	Potential sources of influence of perceived influence on study conduct and conclusions; how these were managed	22
Funding	#21	Sources of funding and other support; role of funders in data collection, interpretation and reporting	22

The SRQR checklist is distributed with permission of Wolters Kluwer © 2014 by the Association of American Medical Colleges. This checklist can be completed online using https://www.goodreports.org/, a tool made by the EQUATOR Network in collaboration with Penelope.ai

For peer review only

BMJ Open

Plain Packaging of Waterpipe Tobacco? A Qualitative Analysis Exploring Waterpipe Smokers' and Non-smokers' Responses to Enhanced versus Existing Pictorial Health Warnings in Egypt

Journal:	*BMJ Open*
Manuscript ID	bmjopen-2018-023496.R1
Article Type:	Research
Date Submitted by the Author:	20-Jul-2018

Complete List of Authors:	Mostafa, Aya; Faculty of Medicine, Ain Shams University, Department of Community, Environmental, and Occupational Medicine<br>Mohammed, Heba Tallah; Faculty of Medicine, Ain Shams University, Department of Community, Environmental, and Occupational Medicine; University of Waterloo, Ontario, School of Pharmacy<br>Mohamed, Wafaa; Faculty of Medicine, Ain Shams University, Department of Community, Environmental, and Occupational Medicine<br>Elhabiby, Mahmoud; Faculty of Medicine, Ain Shams University, Department of Psychiatry Medicine<br>Safwat, Wael; Egyptian Tobacco Control Coalition<br>Labib, Sahar; Tobacco Control Unit, Ministry of Health<br>Aboul Fotouh, Aisha; Faculty of Medicine, Ain Shams University, Department of Community, Environmental, and Occupational Medicine<br>Hoek, Janet; University of Otago, Marketing

**Primary Subject Heading**:	Smoking and tobacco
Secondary Subject Heading:	Public health, Qualitative research, Health policy
Keywords:	Plain packaging, Health warnings, Waterpipe tobacco, Uptake, Cessation, QUALITATIVE RESEARCH

SCHOLARONE™
Manuscripts

RESEARCH ARTICLE

Plain Packaging of Waterpipe Tobacco? A Qualitative Analysis Exploring Waterpipe Smokers' and Non-smokers' Responses to Enhanced versus Existing Pictorial Health Warnings in Egypt

Aya Mostafa[1], Heba Tallah Mohammed[1, 2], Wafaa Mohamed Hussein[1], Mahmoud Elhabiby[3], Wael Safwat[4,5], Sahar Labib[6], Aisha Aboul Fotouh[1], Janet Hoek[7]

[1] Department of Community, Environmental, and Occupational Medicine, Faculty of Medicine, Ain Shams University, Cairo, Egypt

[2] School of Pharmacy, University of Waterloo, Ontario, Canada

[3] Department of Psychiatry Medicine, Faculty of Medicine, Ain Shams University, Cairo, Egypt

[4] Egyptian Tobacco Control Coalition, Cairo, Egypt

[5] Egypt Health Foundation, Cairo, Egypt

[6]Tobacco Control Unit, Ministry of Health, Cairo, Egypt

[7] Departments of Public Health and Marketing, University of Otago, New Zealand

CORRESPONDING AUTHOR

Dr. Aya Mostafa (ORCID: http://orcid.org/0000-0002-2803-2608; Scopus ID: 36010130700)

Department of Community, Environmental, and Occupational Medicine, Faculty of Medicine, Ain Shams University, Abbasia Square, Cairo, Egypt, PO-box 11566 Cairo, Egypt

E-mail: aya.kamaleldin@med.asu.edu.eg Phone: +2 0100 5217379

WORD COUNT Abstract 285, Main text 4,307 (Introduction 773, Methods 1275, Results 1381, Discussion 878).

This manuscript has not been published in another journal, nor is it under review elsewhere.

For peer review only

For peer review only - http://bmjopen.bmj.com/site/about/guidelines.xhtml

ABBREVIATIONS

FCTC — Framework Convention on Tobacco Control

PHWs — Pictorial Health Warnings

WHO — World Health Organization

WT — Waterpipe Tobacco

WTS —Waterpipe Tobacco Smoking

WTPs — Waterpipe Tobacco Packs

For peer review only

For peer review only - http://bmjopen.bmj.com/site/about/guidelines.xhtml

ABSTRACT

Objectives Despite the global increase in waterpipe tobacco smoking (WTS) including in Egypt, few studies have assessed the effectiveness of waterpipe tobacco (WT) health warnings. Egypt has used pictorial health warnings (PHWs) on waterpipe tobacco packs (WTPs) and has rotated these every two years since 2008. We explored in this qualitative study how participants perceived existing PHWs on WTPs, assessed how they interpreted novel plain packaging of WT featuring enhanced PHWs, and probed perceptions of how existing and novel sets would affect uptake or cessation of WTS.

Design We conducted ten qualitative focus groups and ten in-depth interviews. We explored participants' views of the four existing PHWs (occupied 50% of the front and back of WTPs, displayed cancers, and featured colourful fruits and flavors) and four novel PHWs (occupied 80% of the front and back of WTPs, displayed different topical content, with plain packaging). Transcripts were analyzed using thematic analysis.

Setting Rural Menoufia, urban and semi-urban Cairo, Egypt.

Participants 90 waterpipe smokers and non-smokers, males and females, aged 18 years or older.

Outcomes Perceived potential effect on WTS uptake or cessation, probing factors related to PHW content and WTP design.

Results Participants in focus groups and in-depth interviews thought existing WT PHWs elicited affective responses, but found them unclear or unrealistic and thought the colourful packaging detracted from the warnings. In contrast, they thought novel and larger WT PHWs presented in plain packaging might prevent WTS initiation or trigger quit attempts. Participants regarded warnings featuring proximal health risks as most likely to be acceptable.

Conclusions Our exploratory study suggests larger WT PHWs featuring proximal risks and

For peer review only - http://bmjopen.bmj.com/site/about/guidelines.xhtml

presented on plain WTPs could potentially deter experimentation with WT products among non-users and promote cessation among existing users.

The abstract of this study has been presented at the World Conference on Tobacco or Health, 2018 in South Africa: https://doi.org/10.18332/tid/84640.

For peer review only

For peer review only - http://bmjopen.bmj.com/site/about/guidelines.xhtml

ARTICLE SUMMARY

Strengths and limitations of this study

- This is the first qualitative study to explore plain packaging of waterpipe tobacco products in a country that has existing waterpipe tobacco pictorial health warnings.
- We provide novel insights from both non-smokers and smokers into potential policy-relevant outcomes, particularly uptake and cessation of waterpipe tobacco smoking.
- Use of combined focus groups and in-depth interviews as qualitative methods offered rich understanding of perceptions related to waterpipe tobacco labelling, with respect to which contents pictorial health warnings might feature, and how design of waterpipe tobacco packs might be improved.
- Our sample of 90 individuals means we cannot generalize our findings, however we included a variety of participants, and achieved data saturation.
- While we explored projected rather than actual responses to existing and novel waterpipe tobacco pictorial health warnings with plain packaging, our findings could guide future experimental studies and assist policy-makers to improve waterpipe tobacco regulations.

KEY WORDS

Plain packaging, Health warnings, Waterpipe tobacco, Uptake, Cessation, Qualitative research

INTRODUCTION

The introduction of flavoured tobacco and the lack of regulatory policies have seen waterpipe tobacco smoking (WTS) increase globally.[1,2]Misperceptions that WTS is a safe alternative to cigarette smoking may also have contributed to rising waterpipe tobacco (WT) use,[3] even though WTS causes respiratory illnesses, cardiovascular diseases and adverse perinatal outcomes.[4] These factors have helped WTS become more socially acceptable globally, especially among youth[5,6] and women.[7,8]

WT use has extended beyond the East, where it has been present for decades, and is increasingly popular in the West, where WTS rates have reached 10% among some young adult populations in the United States and the UK.[9,10] The WHO Eastern Mediterranean Region (EMR) remains home to the highest WTS rates worldwide;[1,11] in some EMR countries, WTS has surpassed cigarette smoking in females and adolescents.[12,13] Egypt has witnessed a rising trend in WT use; adolescent girls (3.4%)[14] and university students (12.2%)[15] report higher WTS rates than their older counterparts (0.3% in women[14] and 6.2% in men[16]), and rurally-located Egyptian males smoke WT more (7.5%) than men living in urban regions (4.9%).[16] This global surge in WTS makes examining the perceived effectiveness of existing WT control policies important to inform a much needed WT regulatory framework.[17-19]

Applying health warnings to tobacco products can cost-effectively increase public awareness of smoking's risks, increase the likelihood of quitting among smokers, and deter smoking initiation among non-smokers.[20] These outcomes are mediated by several measures of effectiveness that have been organized, based on behavioural theories, within conceptual frameworks of health

For peer review only - http://bmjopen.bmj.com/site/about/guidelines.xhtml

warning impact.[21-24] In line with this evidence, guidelines for implementing Article 11 of the WHO's Framework Convention on Tobacco Control (WHO FCTC) call for on-pack pictorial health warnings (PHWs), and recommend plain packaging and increasing warning size.[25] Egypt, a signatory country to the WHO FCTC, has applied generic PHWs to waterpipe tobacco packs (WTPs).[26] Since 2008, a set of four PHWs has appeared on the bottom half of the front and back of WTPs; these warnings carry the quitline number and rotate every two years.[27] However, WTPs still depict colourful fruits and flavours in brand imagery.[27]

Several observational[28] and experimental studies,[21] suggest plain packaging with larger PHWs[29] could more effectively reduce tobacco smoking through increasing warning salience, making the packaging and smoking less appealing, and reducing misperceptions about product harm,[30,31] especially in non-smokers or non-established smokers.[32] Yet, while this evidence is encouraging, these studies have focussed largely on cigarettes and we know little about how PHWs could reduce non-cigarette tobacco use, particularly WTS.[33]

To our knowledge, only a few studies have examined the impact of WT PHWs: two online surveys from Canada[34] and the United States,[35] three qualitative studies from the UK,[36] Egypt[37] and the EMR,[38] and one recent Egyptian survey.[39] The two online surveys tested hypothetical warnings shown on computer screens rather than on WTPs and examined the effectiveness of text-only versus PHWs.[34,35] Both studies found that PHWs had a modest impact on established waterpipe smokers.[34,35] The UK qualitative study found that when warnings increased in size and packs became less branded, participants felt WTPs were less attractive and warnings were more impactful.[36] EMR study participants reported that PHWs improved respondents' knowledge

For peer review only - http://bmjopen.bmj.com/site/about/guidelines.xhtml

about WTS health hazards.[38] These studies are important but were confined to waterpipe smokers;[34-36,38] while it is important to examine how WT PHWs might pertain to both smokers and non-smokers. The Egyptian qualitative study examined smokers' and non-smokers' responses to placement of PHWs on the waterpipe device. Participants reported this approach could potentially increase salience of WT PHWs, deter initiation of WTS and prompt non-established waterpipe smokers to quit.[37] The Egyptian survey reported that only half of 1048 waterpipe smoker and non-smoker participants thought that existing PHWs on WTPs were visible; they expressed varying views on the effectiveness of WT PHWs across several measures (such as salience, credibility, perceived harm, affective reactions).[39] However, this survey did not examine whether participants perceived existing WT PHWs effective in deterring uptake or quitting of WTS.

Given rising WTS rates in Egypt,[14-16] in 2015 the Tobacco Control Unit in the Egyptian Ministry of Health proposed amending PHW regulations and introducing plain packaging. Specifically, it recommended increasing PHW size to 80% of the pack surface, and removing colours and flavour imagery from tobacco packs. To provide preliminary insights into the potential effects of this approach, we used qualitative methods, which are particularly suited to exploring understudied areas.[40,41] To inform WT labelling policy, we explored how participants perceived existing PHWs on WTPs, assessed how they interpreted a hypothetical scenario where WT was presented in plain packaging and featured enhanced PHWs, and probed perceptions of how existing and novel PHWs would affect uptake or cessation of WTS.

METHODS

For peer review only - http://bmjopen.bmj.com/site/about/guidelines.xhtml

Design

The study comprised ten focus group discussions and ten in-depth interviews that took place in urban and semi-urban regions in Cairo, and a rural area in Menoufia governorate. We utilized both focus groups and in-depth interviews as complementary approaches; focus groups explored participants' interactions and whether and how consensus views evolved while in-depth interviews allowed detailed probing and deeper understanding of participants' views.[40,41] Some sessions were conducted in WTS usage settings, such as cafes, where we observed social and cultural dynamics of WTS and assessed WT PHW's visibility to others.

Our conceptual framework drew on the theory of planned behavior,[42] as outlined in IARC Handbooks of Cancer Prevention, Tobacco Control, Methods for Evaluating Tobacco Control Policies 2008.[24] We explored policy-relevant outcomes with respect to the perceived potential effect on WTS uptake and cessation, and probed factors related to PHW content and WTP design, including salience, affective reactions, perceived harm and credibility. This study was approved by the Ethical Review Board of the Faculty of Medicine, Ain Shams University (FMASU R 10/2015). We used SRQR guidelines for reporting qualitative research.[43]

Sample

Our sample comprised men and women, 18 years of age or older, who lived in rural, urban, and semi-urban locations. We included self-identified waterpipe smokers (exclusive WT or dual users of WT and cigarettes) and non-smokers (non-users of any tobacco product), as we were interested in how warnings could influence WTS initiation as well as cessation. Participants were recruited using snowball sampling,[44]which enabled us to access female waterpipe smokers more

For peer review only - http://bmjopen.bmj.com/site/about/guidelines.xhtml

easily and thus address calls for more research into this hard-to-reach group. We explained the study purpose to people who made contact, invited them to participate in a one-on-one interview or focus group, and then set a meeting date and time. Participants did not discernibly differ by type of interview chosen.

In total, 90 individuals participated, including 80 in homogenous focus groups (with respect to age, gender, smoking status) with 6–8 individuals per group, and 10 in in-depth interviews **(see Supplementary Table 1)**. As WT use in Egypt is generally higher among males,[16] more males participated in our sessions. **Supplementary Table 2** contains details of participants' demographic characteristics.

Tools

Interview Guide

We developed the interview guide in Egyptian colloquial Arabic and incorporated qualitative measures used to assess tobacco labelling policies.[24] We pilot tested the interview guide for clarity and comprehensiveness, tested the appropriateness of our prompts and questions after the pilot sessions, and made modifications following discussions with the research team. We used the same guide with focus groups and in-depth interviews and probed participants' experiences of WTS, their knowledge of WT PHWs, their views on the existing and novel PHWs on WTPs, and their perceptions of placing PHWs on waterpipe devices **(see Supplementary for interview guide).** In this article, we focus on discussing PHWs on WTPs.

Pictorial Health Warnings on Waterpipe Tobacco Packs

For peer review only - http://bmjopen.bmj.com/site/about/guidelines.xhtml

The existing PHWs depicted cancers of lung, throat, mouth, and face**,** covered 50% of the lower surface of the front and back of the WTP against a colourful background depicting fruit and flavour imagery. The warnings included pictures, generic text and the quitline number **(Figure 1A)**.

We adapted novel PHWs from a health warning database[45] and followed WHO FCTC recommendations for plain packaging[25] and WHO's publication on Evidence, Design and Implementation of Plain Packaging [46] building on the proposal of the Tobacco Control Unit of the Egyptian Ministry of Health; the PHW thus covered 80% of the upper surface of the front and back of the WTP against a dark uniform plain background not depicting any fruit or flavour imagery, with the remaining 20% depicting only the brand name in standardised font. The novel PHWs included pictures, text and the quitline number. Dark plain packs are perceived as more harmful[46] and culturally as more negative (when compared with the bright background colors of the existing WTPs) and we therefore used a drab dark brown colour (similar to that used in Australian packaging) on novel WTPs. Feedback from pilot testing indicated that the dark background color contrasted well with the white color of the textual message and the yellow background of the quitline number, making both more clear and salient. We applied newly designed PHWs to used WTPs to promote authenticity. We pilot tested health warnings messages with corresponding images for clarity and comprehension: ("Smoking kills," "Smoking causes lung cancer", "Smoking causes clotting of blood vessels", "Smoking causes blindness", "Smoking during pregnancy harms fetus", and "Don't let your children inhale your smoke"). The warnings used were selected following discussions amongst the research team and feedback from pilot sessions, and reflected the best available evidence on WTS health

For peer review only - http://bmjopen.bmj.com/site/about/guidelines.xhtml

outcomes.[47] WTS share common harms with those caused by cigarette smoking.[4] We sought topical content showing less severe harms than those depicted in existing PHWs. The four novel warnings selected included the effect of smoking during pregnancy on the fetus, effects of peripheral vascular diseases affecting the feet and eye, and effects on teeth and gums **(Figure 1 B)**. Although it was important to adapt the textual message to be waterpipe-specific, we did not test this in the study reported here; this was assessed separately in another study of our research project.

Data Collection

Data were collected from October 2015 to February 2016 at the Faculty of Medicine, Ain Shams University in Cairo (five focus groups and five in-depth interviews), and at participants' homes or in cafés for those in rural and semi-urban areas (five focus groups and five in-depth interviews). All participants received an information letter explaining the study and were asked to provide verbal consent prior to each discussion or interview commencing. Participants were advised their data and identity would be confidential, and told they could withdraw from the study at any time.

Each focus group or interview was moderated by two of the co-authors (AM, WS, ME, and WM) and audio-recorded; each session was about one hour long. The facilitator and note taker regularly switched roles to promote reflection, and the wider team critically reflected on the interviews during team meetings. The facilitators followed standard procedures when discussing the interview guide topics and introduced the PHW stimuli when the relevant topic was opened for discussion. No further sessions were scheduled once data saturation had been reached, i.e. no new themes were being generated during the discussions.[48]

For peer review only - http://bmjopen.bmj.com/site/about/guidelines.xhtml

Analysis

Two authors independently transcribed verbatim the recorded sessions in their original language then translated these into English before comparing the two transcripts to ensure inclusivity and accuracy (WH, HM); a third author (AM) back translated the transcripts independently for validity purposes; any discrepancies were resolved through discussion. Considering the identical aims and topics explored, data from focus groups and interviews were analyzed together. We analysed the data using a thematic approach.[49,50] We coded transcripts as the study progressed using a three-phase process that began by organizing ideas in relation to the research questions, then involved independent reviews of transcripts to identify preliminary themes and create an initial coding list.[51] We finally independently refined this list (AM, AA), added new codes where appropriate, and developed broader themes, that one author (HM) then reviewed across all cases. We resolved minor inconsistencies during discussion sessions and after extensive reviews of transcripts before we finalized the themes and subthemes **(see Supplementary Table 3)**. In this article, we focus on policy-relevant outcomes relevant to PHWs on WTPs as described above in methods “design”.

Patient and Public Involvement

Patient and public were not involved in the development of the research question and outcome measures, the design, recruitment and conduct of the study. The results of this study will be disseminated to study participants via newsletters and social media outlets.

RESULTS

For peer review only - http://bmjopen.bmj.com/site/about/guidelines.xhtml

The 90 participants in focus groups and interviews comprised more males (72.2%) than females (27.8%) and participants' mean age was 33.4 ±11.6 **(see Supplementary Table 2)**. We identified the overall themes: warning label content and pack design features, and discuss these in relation to WTS uptake and cessation comparing throughout existing and novel sets. During analysis of the transcripts, we did not detect differences between the focus group and individual interview data. Therefore, we report below results from both focus groups and individual interviews. We cite exemplar quotations below and provide a more detailed set of quotations in **Supplementary Table 4**, where we also indicate the gender, age group, smoking status, location of participants, and source of quotations.

Most participants were aware of warning labels on WTPs and reported seeing these when purchasing or preparing their tobacco. However, those using waterpipes in cafes or were less likely to see WTPs as WT was prepared out of their sight *"I was downtown and we saw smokers in cafes but shisha is always served ready…I never saw the packs" (Female non-smoker,>25y,semi-urban,FGD).*

Warning label content – perceived likely effect of existing and novel PHWs

Participants who were aware of existing WTP warnings recalled these as disturbing; however, several felt these warnings had limited impact. Many recalled the lung cancer PHW and the text "smoking is hazardous to health and causes death" as the most believable warning, yet also the least impactful, because of wearout: *"…the other warnings like the lung cancer one…people got used to them after a while" (Male smoker,>25y,rural,IDI).* Participants found the existing warnings, which all featured cancer-related harms, frightening and disgusting, and non-smokers, particularly, avoided looking at them. Nonetheless, several questioned the harms existing PHWs

For peer review only - http://bmjopen.bmj.com/site/about/guidelines.xhtml

featured and saw these as exaggerated: *"I don't want to look at it from near or far…they want to send us a message that it is harmful but in an awful and overstating way" (Female smoker,<25y, urban,FGD).*

Some also found existing warnings difficult to understand: *"It looks like a bad thing but it is not clear what it is" (Female non-smoker,>25y,semi-urban,FGD)*. These participants saw existing PHWs as unconvincing and exaggerated as they had not seen such conditions in real life and sometimes knew people who had smoked for many years with apparent impunity: *"I have seen the warning on the packs but I have never seen anything such as that in real life…I know a person who has been smoking since the eighties and nothing happened to him" (Male smoker,>25y,rural,FGD).*

Participants often denied risks associated with occasional smoking; one exempted himself from harm on the grounds that he did not smoke heavily and had seen no direct evidence of the harms presented in PHWs: *"I don't think I can be affected by smoking, because I don't smoke heavily; only once a day, besides...we have never seen the conditions in the warnings in real life" (Male smoker,>25y,rural,IDI).* Several thought harms would not occur until they were older, and believed that, if and when they experienced these, they could quit.

By contrast, most participants favoured the new PHWs, which they first viewed during the interview sessions. They found these clearer, more understandable and realistic than existing PHWs, and more likely to capture their attention. Participants also commented that the new PHWs were easily understood even without text and thus likely to be effective among people

For peer review only - http://bmjopen.bmj.com/site/about/guidelines.xhtml

with varying literacy: *"This one is more realistic for its purpose and understandable; even without any text…it is a more convincing warning" (Female non-smoker,>25y,semi-urban,FGD).*

Specifically, participants found the new warnings featuring more immediate, proximal risks had the strongest perceived impact, particularly those showing harmful effects on teeth. Female participants also felt strongly affected by the PHW illustrating harm to unborn babies, which forced them to confront the harm they imposed on others: *"The baby warning is effective because it is not about me anymore…This is something way more important than me…I fear for my kid more than I fear for myself" (Female non-smoker,>25y,semi-urban,FGD).* However, some continued to see images showing potential harms they may experience as overstated and unlikely to happen in the near future: *"This didn't happen before to anyone (foot warning)…we're still young…we won't get this" (Female smoker,<25y,urban,FGD).*

Although participants saw the new PHWs as more effective, they suggested improvements to the warning content. In line with earlier comments about perceived exaggeration, they sought greater credibility: *"It is very important that you convince me…put something there that I'll believe" (Male smoker,>25y,semi-urban,IDI).* Some suggested presenting testimonials: "*Show smokers live people who were damaged because of shisha smoking and others who quit and improved*" *(Male non-smoker,>25y,rural,FGD)* and others recommended illustrating the effects WTS has on women by featuring relevant cancers and congenital diseases: "*Direct warnings addressing women like cancer of the breast or the uterus or congenital anomalies to the fetus" (Female non-smoker,>25y,semi-urban,FGD).* Some also thought PHWs targeting women could encourage them to persuade their partners to quit.

For peer review only - http://bmjopen.bmj.com/site/about/guidelines.xhtml

Several participants thought PHWs should target youth before they start smoking and suggested warnings showing how WTS smokers' social relationships (e.g., sexual dysfunction warnings) would have high impact: *"We want a real effect that already happened…like for example the side effects on sexual functioning…that will definitely affect smokers" (Male non-smoker,>25y,rural,FGD).*

In addition, participants also suggested printing text warnings including details of the hazardous ingredients of smoke, with more information on health risks and cessation options inserted on the inside or outside of the WTP, together with external PHWs would promote cessation behaviour, and enhance the impact of novel warnings.

Design Features of WTPs – perceived likely effect of existing and novel PHWs

As well as responding to the different warning content, participants also noted that the different design elements used in the novel PHWs had improved the impact these had. Several commented on how bright colours and fruit imagery deflected attention away from PHWs and promoted experimentation: *"The peach drawing is appetizing…* [and] *drawing attention away from the warning…better put the warning on the top! The pack should be dark…this colour is very bright… just like bonbon packs" (Female non-smoker,>25y,semi-urban,FGD).* They found it difficult to associate fruit flavours with harm and thought the images invited trial: *"The pictures and the smell of fruits make a passerby want to try them all"(Female non-smoker,>25y,semi-urban,IDI).*

For peer review only - http://bmjopen.bmj.com/site/about/guidelines.xhtml

By contrast, participants thought the plain background, contrasting colours, absence of fruit and flavour images, and larger warning images shown on the proposed new PHWs increased impact, reduced distraction, and encouraged participants to look more closely at the pack. One noted: "*Here the picture is bigger and the text has a clear message…together with this dark colour…it makes me focus only on the warning…all this makes it more effective" (Male smoker,<25y,urban,FGD)*. Together, the altered content and enhanced design attributes increased participants' perceptions of the impact the novel PHWs would have.

Overall, most waterpipe smoker and non-smoker participants thought PHWs would deter non-smokers from trying WTS but were less optimistic about the effects on smokers. One noted: "*If we (non-smokers) lusted to smoke it and saw pictures like these…we won't smoke, but those who do actually smoke already would be indifferent" (Male non-smoker,>25y,urban,FGD).* Smokers themselves also felt PHWs had less effect because they had become accustomed to seeing the images: *"I used to think about the hazards a lot when the pictures first appeared…then I got used to them…I don't pay them attention anymore" (Male smoker,<25y,semi-urban,IDI).* Others reported using stickers to obscure PHWs or avoiding packs with PHWs they found particularly confronting: *"I avoid buying the picture of the tongue in particular* (referring to the mouth cancer warning)" *(Male smoker,<25y,semi-urban,FGD)*

As with the current PHWs, most waterpipe smoker and non-smoker participants thought the proposed PHWs would have a stronger effect on non-smokers than on long-term smokers, though some indicated they would avoid some warnings and may reduce their WTS: *"If I go to buy moassel (waterpipe tobacco) and found this pack, I'll go to another shop to buy another*

For peer review only - http://bmjopen.bmj.com/site/about/guidelines.xhtml

one…if I don't find a picture that makes me comfortable…I won't smoke that day…but if they're all like this…I guess I'll try to quit…or… at least decrease my habit" (Male smoker,>25y,rural,IDI). In general, participants thought that the new PHW set had greater potential to deter WTS, especially among new smokers: *"If I'll smoke and saw it… for sure I won't smoke at the moment…it's disgusting" (Male non-smoker,<25y,rural,FGD).*

DISCUSSION

Our qualitative study found that participants privileged the short-term benefits they received over the longer-term risks they faced. They saw existing PHWs as less likely to influence long-term smokers, [52] especially if they had not experienced any health effects. This finding is consistent with other studies that found age is negatively associated with perceived risks of smoking and with attention to either graphic or text warnings on tobacco packages.[53]

However, the existing and novel PHWs tested appeared more likely to influence non-smokers and less-experienced smokers by creating awareness of the health risks WTS presents. These findings are in line with previous research[32] and address calls for research into the effects of tobacco packaging on smoking uptake.[54] We also provide preliminary evidence that presenting WT in plain packaging could deter non-smokers from experimenting WTS. The existing and the novel PHWs differed in three main ways: the topical imagery content, the size of the warning, and the pack design. We explain below how policymakers should consider these three elements when adopting or amending regulations for PHWs on WTPs.

Although existing PHWs induced strong negative emotional reactions, several participants

For peer review only - http://bmjopen.bmj.com/site/about/guidelines.xhtml

viewed these warnings as exaggerated and felt the health risk depicted was unlikely to occur. While PHWs that arouse fear may increase risk perceptions, they did not necessarily promote greater message acceptance,[55-57] Our findings also show a complex relationship between the emotional response elicited and the salience and perceived impact of a message. The PHW depicting oral harm was minimally disturbing, yet participants saw it as the strongest and the most salient warning; by contrast, participants regarded the confronting vascular harm PHW as less effective because the condition was less salient and seen as more distal. Our findings thus support earlier studies analysing the relationship between risk perception, believability, and temporal distance.[58]

Young adults and women found gender- and age-specific messages more persuasive,[59] suggesting strategies targeting these demographic groups are crucially important[60] For example, messages about negative health effects from passive smoking, sexual dysfunction and intimacy, miscarriages, and harmful effects on infants and children were considered more persuasive among younger adults and women than other messages.

Another plausible approach suggested by participants was to develop a visible and sensible message in both text and graphic formats that target waterpipe smokers with detailed cessation information inserted on the WTP. Combined text and graphic warnings elicit adverse reactions to smoking among non-smokers and smokers,[61] especially on the sustainability of quit behaviour.[62] In line with these suggestions, the design of novel warnings helped make the cessation quit line more prominent by the contrasting dark plain background.

For peer review only - http://bmjopen.bmj.com/site/about/guidelines.xhtml

We also found specific design elements that could inform future PHW development. Some participants thought plain packs increased the salience and effectiveness of the warnings, and reduced the appeal of the packaging and misperceptions of harm. These findings are consistent with the literature on plain packaging.[46,54] Evidence that flavoured cigarettes reduce harm perceptions appears relevant to WT[63,64] as participants noted that images of fruits or appealing flavours attracted them to WTS and deflected their attention from warning information. Furthermore, image clarity and size enhanced acceptance and risk perception among participants, a finding also reported by Jawad and colleagues.[36]

Strengths and Limitations

To our knowledge, this is the first qualitative study to assess smokers' and non-smokers' awareness and acceptance of currently used and novel WT PHWs with plain packaging. Our novel warnings simulated how PHWs appear (or could appear) on packs and devices, we explored projected rather than real-life responses. Experimental work could estimate the likely impact of our new label designs, including more targeted PHWs, on waterpipe smokers' risk perceptions, attitudes and likely cessation responses. Future research could also develop a more comprehensive analysis of factors motivating and reinforcing WTS uptake. Given our findings that PHWs have the potential to reduce WTS initiation, it is also important to test whether the PHW themes we developed could be used in wider health promotion campaigns to reduce the appeal and perceived acceptability of WTS. Future research could explore how WT warnings featuring waterpipe-specific messages would affect awareness and perceptions of WTS.

Our small sample means we cannot generalize our findings, though we note a sample of 90 individuals is still substantial, and saturation had been reached in the responses received. Despite

For peer review only - http://bmjopen.bmj.com/site/about/guidelines.xhtml

these limitations, our study provides novel insights into the factors supporting WTS uptake, suggests themes PHWs could feature, and outlines how PHWs' format could be improved. We recognize this work programme faces challenges, given the limited resources and capacity in low- and middle-income countries; nonetheless, our findings represent an important step in supporting a comprehensive regulatory framework that reduces WTS and the harm this form of tobacco use causes.

Conclusions

This exploratory study suggests that PHWs on WTPs have the potential to reduce uptake and cue quit attempts but might be more effective if PHWs used more impactful designs. Specifically, we provide preliminary evidence that enhanced PHWs using contrasting background colours and plain packaging, offering no association to fruits or flavours, targeting age and gender, and displaying proximal health risks, might enhance both warning impact and risk perception. These alternative designs could be further developed and tested in other studies. The findings offer policymakers designing and implementing health warnings on WT products clearer evidence on which to base their decisions.

For peer review only

For peer review only - http://bmjopen.bmj.com/site/about/guidelines.xhtml

ACKNOWLEDGEMENTS We thank the participants of this research and Joe Petrik for copyediting a previous version of this article. The PHWs presented in this study were used after signing a written partnership agreement between the Tobacco Control Unit in the Egyptian Ministry of Health and the research team. The abstract of this study has been presented at the World Conference on Tobacco or Health, 2018 in South Africa: https://doi.org/10.18332/tid/84640.

AUTHOR CONTRIBUTORS AM conceptualized and designed the study, developed its tools, and wrote the first draft of this article. AM, ME, WS, WMH carried out the study. SL provided approval for using PHWs. WMH and HM transcribed the data. AA, HM, AM and JH analyzed the data. AM, HM, AA and JH further developed the paper. All authors critically revised contents and provided final approval on the submitted manuscript.

FUNDING This work was carried out with the aid of a grant from the International Development Research Center, Ottawa, Canada through the American University of Beirut, the Tobacco Control Research Group, to study waterpipe tobacco smoking prevention and intervention programs in the region, as a part of the project "Shaping Research for Health in the Arab World: A Systems and Network Approach to Advance Knowledge, Inform Policy, and Promote Public Health" (Grant 106981-001)

COMPETING INTERESTS None declared.

ETHICS APPROVAL This study was approved by the Research Ethics Committee of Faculty of Medicine, Ain Shams University (FMASU R 10/2015).

CHECKLIST FOR REPORTING GUIDELINES We used SRQR guidelines for reporting

For peer review only - http://bmjopen.bmj.com/site/about/guidelines.xhtml

qualitative research.

DATA SHARING STATEMENT No additional data are available.

For peer review only

For peer review only - http://bmjopen.bmj.com/site/about/guidelines.xhtml

REFERENCES

1. Maziak W, Ben Taleb Z, Bahelah R, et al. The global epidemiology of waterpipe smoking. *Tob Control* 2015,24(1):i3-i12. doi:10.1136/tobaccocontrol-2014-051903.
2. Jawad M, Charide R, Waziry R, et al. The prevalence and trends of waterpipe tobacco smoking: A systematic review. *PLoS ONE* 2018; 13(2): e0192191. doi.org/10.1371/journal.pone.0192191.
3. Jawad M. Waterpipe Tobacco Smoking: A less Harmful Alternative? In: Loddenkemper R, Kreuter M (eds): The Tobacco Epidemic, ed 2, rev. and ext. Prog Respir Res. Basel, Karger, 2015,42:252-257. doi:10.1159/000369505.
4. Waziry R, Jawad M, Ballout RA, et al. The effects of waterpipe tobacco smoking on health outcomes: an updated systematic review and meta-analysis. *Int J Epidemiol* 2017;46(1):32-43. doi:10.1093/ije/dyw021.
5. Soule EK, Lipato T, Eissenberg T. Waterpipe tobacco-smoking: a new smoking epidemic among the young? *Curr Pulmonol Rep* 2015;4(4):163-172. doi:10.1007/s13665-015-0124-6.
6. Akl EA, Ward KD, Bteddini D, et al. The allure of the waterpipe: a narrative review of factors affecting the epidemic rise in waterpipe smoking among young persons globally. *Tob Control* 2015;24:i13–i21. doi:10.1136/tobaccocontrol-2014-051906.
7. Akl EA, Gunukula SK, Aleem S, et al. The prevalence of waterpipe tobacco smoking among the general and specific populations: a systematic review. *BMC Public Health* 2011;11:244. doi:10.1186/1471-2458-11-244.
8. Khalil J, Afifi R, Fouad FM, et al. Women and Waterpipe Tobacco Smoking in the Eastern Mediterranean Region: Allure or Offensiveness: *Women & Health* 2013;53(1):100-116. doi:10.1080/03630242.2012.753978.

For peer review only - http://bmjopen.bmj.com/site/about/guidelines.xhtml

9. Cobb C, Ward KD, Maziak W, et al. Waterpipe tobacco smoking: An emerging health crisis in the United States. *Am J Health Behav* 2010;34(3),275–285. PMCID: PMC3215592.

10. Grant A, Morrison R, Dockrell RJ. The Prevalence of waterpipe (Shisha, Narghille, Hookah) use among adults in great britain, and factors associated with waterpipe use: data from cross-sectional online surveys in 2012 and 2013. *Nicotine Tob Res* 2014;16(7): 931–938. doi:10.1093/ntr/ntu015.

11. Maziak W, Nakkash R, Bahelah R, et al. Tobacco in the Arab world: old and new epidemics amidst policy paralysis. *Health Policy Plan* 2013;29:784–94. doi.org/10.1136/tobaccocontrol-2014-051903.

12. Mostafa A, El Houssinie M, Aboul Foutouh A. Multiple Tobacco Use among Young Adult Waterpipe Smokers in Egypt. *East Mediterr Health J* 2018;24(x):xxx–xxx. https:// doi.org/2018.24.2.XX. In press.

13. Jawad M, Lee JT, Millett C. Waterpipe Tobacco Smoking Prevalence and Correlates in 25 Eastern Mediterranean and Eastern European Countries: Cross-Sectional Analysis of the Global Youth Tobacco Survey. *Nicotine Tob Res* 2016;18(4):395-402. doi:10.1093/ntr/ntv101.

14. El Awa F, Fouad H, El Naga RA, et al. Prevalence of tobacco use among adult and adolescent females in Egypt. *East Mediterr Health J* 2013;19(8):749–54. PMID:24975361.

15. Shisha and smokeless tobacco use among university students in Egypt: prevalence, determinants, and economic aspect. A joint report by the Egyptian Ministry of Health and Population and the World Health Organization 2014. http://applications.emro.who.int/dsaf/EMROPUB_2014_EN_1752.pdf?ua=1&ua=1 (accessed May 2017).

For peer review only

For peer review only - http://bmjopen.bmj.com/site/about/guidelines.xhtml

16. World Health Organization: Global Adult Tobacco Survey: Egypt Country Report 2009. http://www.who.int/tobacco/surveillance/gats_rep_egypt.pdf (accessed September 2017).

17. World Health Organization Framework Convention on Tobacco Control: Control and prevention of waterpipe tobacco products (document FCTC/COP/7/10). In: Conference of the parties to the WHO framework convention on tobacco control, seventh session, Delhi, India, 7-12 November 2016. World Health Organization, Geneva. http://www.who.int/fctc/cop/cop7/FCTC_COP_7_10_EN.pdf (accessed May 2017).

18. Jawad M, El Kadi L, Mughrabil S, et al. Waterpipe tobacco smoking legislation and policy enactment: a global analysis. *Tob Control* 2015;24(1):i60-i65. doi:10.1136/tobaccocontrol-2014-051911.

19. Salloum RG, Asfar T, Maziak W. Toward a Regulatory Framework for the Waterpipe. Am *J Public Health* 2016;106(10):1773-7. doi:10.2105/AJPH.2016.303322.

20. Hammond D. Health warning messages on tobacco products: a review. *Tob Control* 2011;20(5):327–337. doi.org/10.1136/tc.2010.037630.

21. Noar SM, Hall MG, Francis DB, et al. Pictorial cigarette pack warnings: a meta-analysis of experimental studies. *Tob Control* 2016;25(3):341-54. doi:10.1136/tobaccocontrol-2014-051978.

22. Yong HH, Borland R, Thrasher JF, et al. Mediational pathways of the impact of cigarette warning labels on quit attempts. *Health Psychol* 2014;33(11):1410–20. doi: 10.1037/hea0000056.

23. Emery LF, Romer D, Sheerin KM, Jamieson KH, Peters E. Affective and Cognitive Mediators of the Impact of Cigarette Warning Labels. *Nicotine Tob Res* 2014;16(3):263–9. doi.org/10.1093/ntr/ntt124.

For peer review only - http://bmjopen.bmj.com/site/about/guidelines.xhtml

24. IARC Handbooks of Cancer Prevention, Tobacco Control. Methods for Evaluating Tobacco Control Policies 2008; 12. Lyon, France. http://www.iarc.fr/en/publications/pdfs-online/prev/handbook12/Tobacco_vol12.pdf (accessed April, 2015).

25. World Health Organization. Guidelines for implementation of Article 11 of the WHO Framework Convention on Tobacco Control (Packaging and labelling of tobacco products) 2008. http://www.who.int/fctc/guidelines/article_11.pdf?ua=1 (accessed April, 2015).

26. Mostafa A, Kashiwabara M. Tobacco packaging and labelling policies in countries of the Eastern Mediterranean and Western Pacific Regions: Post-deadline assessment of the time-bound measures of WHO FCTC Article 11. *Tobacco Prevention & Cessation* 2016;2(November):78. doi:10.18332/tpc/66793.

27. World Health Organization: Tobacco control country profiles 2017. Egypt. http://www.who.int/tobacco/surveillance/policy/country_profile/egy.pdf?ua=1 (accessed 15 December 2017).

28. Stead M, Moodie C, Angus K, et al. Is Consumer Response to Plain/Standardised Tobacco Packaging Consistent with Framework Convention on Tobacco Control Guidelines? A Systematic Review of Quantitative Studies. *PLoS ONE* 2013;8(10): e75919. doi:10.1371/journal.pone.0075919.

29. Wakefield M, Coomber K, Zacher M, et al. Australian adult smokers' responses to plain packaging with larger graphic health warnings 1 year after implementation: results from a national cross-sectional tracking survey. *Tob Control* 2015;24(2):ii17–ii25. doi:10.1136/tobaccocontrol-2014-052050.

30. Durkin S, Brennan E, Coomber K, et al. Short-term changes in quitting-related cognitions and behaviours after the implementation of plain packaging with larger health warnings:

For peer review only - http://bmjopen.bmj.com/site/about/guidelines.xhtml

findings from a national cohort study with Australian adult smokers. *Tob Control* 2015;24:ii26-ii32. doi:10.1136/tobaccocontrol-2014-052058.

31. Mays D, Niaura RS, Evans WD, et al. Cigarette packaging and health warnings: the impact of plain packaging and message framing on young smokers. *Tob Control* 2015;24(e1):e87-92. doi: 10.1136/tobaccocontrol-2013-051234.
32. Munafo MR, Roberts N, Bauld L, et al.: Plain packaging increases visual attention to health warnings on cigarette packs in non-smokers and weekly smokers but not daily smokers. *Addiction* 2011;106(8):1505-10. doi:10.1111/j.1360-0443.2011.03430.x
33. Ross JC, Noar SM, Sutfin EL: Systematic Review of Health Communication for Non-Cigarette Tobacco Products. *Health Communication* 2017; doi:10.1080/10410236.2017.1407274.
34. Mohammed H T. The efficacy of viewing health warnings on shisha smoking among shisha smokers. https://uwspace.uwaterloo.ca/bitstream/handle/10012/7419/Mohammed_HebaTallah.pdf?sequence=1 (accessed September, 2017).
35. Islam F, Salloum RG, Nakkash R, et al.: Effectiveness of health warnings for waterpipe tobacco smoking among college students. *Int J Public Health* 2016;61(6):709-715. doi: 10.1007/s00038-016-0805-0.
36. Jawad M, Bakir A, Ali M, et al.: Impact of Waterpipe Tobacco Pack Health Warnings on Waterpipe Smoking Attitudes: A Qualitative Analysis among Regular Users in London. *BioMed Res Int* 2015:745865.doi: 10.1155/2015/745865.
37. Mostafa A, Mohammed HT, Hussein WM, et al. Would placing pictorial health warnings on waterpipe devices reduce waterpipe tobacco smoking? A qualitative exploration of Egyptian

For peer review only - http://bmjopen.bmj.com/site/about/guidelines.xhtml

waterpipe smokers' and non-smokers' responses. *Tob Control* 2018 Published Online First: 06 July 2018. doi: 10.1136/tobaccocontrol-2018-054494.

38. Salloum RG, Abu-Rmeileh N, Hamadeh R, et al.: Policy-Relevant Context of Waterpipe Tobacco Smoking among University Students in Six Countries Across the Eastern Mediterranean Region: A Qualitative Study. *Asian Pac J Cancer Prev* 2017;18(9): 2533-2540. doi:10.22034/APJCP.2017.18.9.2533.
39. Mostafa A, Mohammed HT. Graphic health warnings and their best position on waterpipes: A cross-sectional survey of expert and public opinion. *Tobacco Prevention & Cessation* 2017; 3(June):116. doi: 10.18332/tpc/70873.
40. Jones R: Why do qualitative research? *BMJ* 1995; 311(6996):2. PMCID: PMC2550075.
41. Pope K, Mays R: Qualitative research: Reaching the parts other methods cannot reach: An introduction to qualitative methods in health and health services research. *BMJ* 1995; 311 (6996):42-5. PMCID: PMC2550091.
42. Ajzen I. The theory of planned behavior. Organ Behav Hum Decis Process 1991;50(2):179–211. https://doi.org/10.1016/0749-5978(91)90020-T.
43. O'Brien BC, Harris IB, Beckman TJ, et al. Standards for reporting qualitative research: a synthesis of recommendations. *Acad Med* 2014;89(9):1245-1251.
44. Feldman MS, Bell J, Berger MT. Gaining Access: A Practical and Theoretical Guide for Qualitative Researchers, Altamira Press 2003, Oxford, UK.
45. World Health Organization Framework Convention on Tobacco Control. Health Warnings Database: http://www.who.int/tobacco/healthwarningsdatabase/en/ (accessed April 2015).

For peer review only - http://bmjopen.bmj.com/site/about/guidelines.xhtml

46. World Health Organization. Evidence, Design and Implementation of Plain Packaging at: http://apps.who.int/iris/bitstream/10665/207478/1/9789241565226_eng.pdf?ua=1 (accessed December 2016).

47. Akl EA, Gaddam S, Gunukula SK, et al. The effects of waterpipe tobacco smoking on health outcomes: A systematic review. *Int J Epidemiol* 2010;39:834–857. doi:10.1093/ije/ dyq002.

48. Walker JL. Research column. The use of saturation in qualitative research. *Can J Cardiovasc Nurs* 2012;22:37-46. PMID: 22803288.

49. Braun V, Clarke V. Using thematic analysis in psychology. *Qualitative Research in Psychology 2006;* 3(2,): 77–101. doi:10.1191/1478088706qp063oa.

50. Pope C, Ziebland S, Mays N: Qualitative research in health care: Analysing qualitative data. *BMJ* 2000;320(7227):114–116. PMCID: PMC1117368

51. Boyatzis RE: Transforming qualitative information: thematic analysis and code development California: Sage Publications; 1998.

52. White V, Bariola E, Faulkner A, et al. Graphic Health Warnings on Cigarette Packs: How Long Before the Effects on Adolescents Wear Out? *Nicotine Tob Res* 2015;17(7):776-83. doi:10.1093/ntr/ntu184.

53. Robert ME, Peters E, Ferketich AK, et al. The Age-related Positivity Effect and Tobacco Warning Labels. *Tob Regul Sci* 2016;2(2):176-185. doi:10.18001/TRS.2.2.8.

54. McNeill A, Gravely S, Hitchman SC, et al. Tobacco packaging design for reducing tobacco use. *Cochrane Database of Systematic Reviews* 2017, Issue 4. Art. No.: CD011244. doi:10.1002/14651858.CD011244.pub2.

For peer review only - http://bmjopen.bmj.com/site/about/guidelines.xhtml

55. Peters GJ, Ruiter RA, Kok G. Threatening communication: A critical re-analysis and a revised meta-analytic test of fear appeal theory. *Health Psychology Review* 2013;7(1):S8–S31. doi:10.1080/17437199.2012.703527.

56. Thrasher J, Swayampakala K, Borland R, et al. Influences of Self-Efficacy, Response Efficacy, and Reactance on Responses to Cigarette Health Warnings: A Longitudinal Study of Adult Smokers in Australia and Canada. *Health Commun* 2016;31(12):1517-1526. doi:10.1080/10410236.2015.1089456.

57. Droulers O, Gallopel-Morvan K, Lacoste-Badie S, et al. The influence of threatening visual warnings on tobacco packaging: Measuring the impact of threat level, image size, and type of pack through psychophysiological and self-report methods. *PLoS One* 2017;12(9):e0184415. doi:10.1371/journal.pone.0184415.

58. Hoek J, Hoek-Sims A, Gendall P. A qualitative exploration of young adult smokers' responses to novel tobacco warnings. *BMC Public Health* 2013;13:609. doi: 10.1186/1471-2458-13-609.

59. Jaber R, Madhivanan P, Khader Y, et al. Predictors of waterpipe smoking progression among youth in Irbid, Jordan: A longitudinal study (2008-2011). *Drug Alcohol Depend* 2015;153:265-70. doi:10.1016/j.drugalcdep.2015.05.008.

60. Koval J, Aubut J, Pederson L, et al. The Potential Effectiveness of Warning Labels on Cigarette Packages. The Perceptions of Young Adult Canadians. *Can J Public Health* 2005; 96(5):354. doi:10.1016/j.drugalcdep.2015.05.008.

61. Singh A, Owusu-Dabo E, Britton J, et al. "Pictures don't lie, seeing is believing": exploring attitudes to the introduction of pictorial warnings on cigarette packs in Ghana. *Nicotine Tob Res* 2014;16(12):1613-9. doi:10.1093/ntr/ntu127.

For peer review only - http://bmjopen.bmj.com/site/about/guidelines.xhtml

62. Thrasher J, Swayampakala K, Cummings M, et al. Cigarette package inserts can promote efficacy beliefs and sustained smoking cessation attempts: A longitudinal assessment of an innovative policy in Canada. *Preventive Medicine* 2016; 88:59–65. doi:10.1016/j.ypmed.2016.03.006.

63. Rising J, Alexander L. Marketing of menthol cigarettes and consumer perceptions. *Tob Induc Dis* 2011;9(1): S2. doi:10.1186/1617-9625-9-S1-S2.

64. Hammond D, Parkinson C. The impact of cigarette package design on perceptions of risk. *Journal of Public Health* 2009;31(3):345-53. doi:10.1093/pubmed/fdp066.

For peer review only

For peer review only - http://bmjopen.bmj.com/site/about/guidelines.xhtml

Figure 1. Pictorial Health Warnings used in this study

For peer review only

For peer review only - http://bmjopen.bmj.com/site/about/guidelines.xhtml

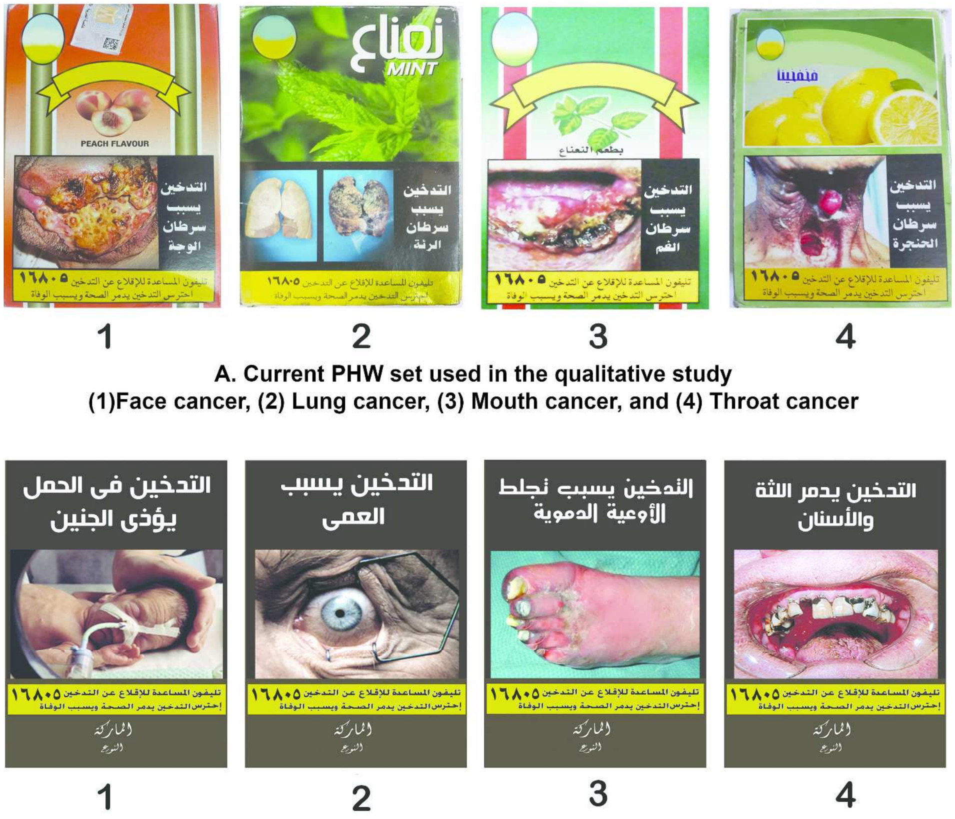

B. Alternative PHW set used in the qualitative study
(1)Fetal harm, (2) Blindness, (3) Blood vessel clotting, (4) Tooth and gum decay
Figure 1. PHWs used in the study

Figure 1. Pictorial Health Warnings used in this study

297x300mm (300 x 300 DPI)

Supplementary

Table 1. Total number of interviews and focus group discussions conducted by demographic profile of participants

	FGDs (10, total participants=80)				IDIs (10)			
	Males (7)		Females (3)		Males (7)		Females (3)	
Age	S	NS	S	NS	S	NS	S	NS
	(5)	(2)	(2)	(1)	(6)	(1)	(2)	(1)
18-24	2	1	1		3	1	1	
25+	3	1	1	1	3		1	1

FGDs=Focus group discussions, IDIs=In-depth interviews, S=Waterpipe smoker, NS=Non-smoker

For peer review only

For peer review only - http://bmjopen.bmj.com/site/about/guidelines.xhtml

Table 2. Sociodemographic characteristics and waterpipe smoking status of participants

	N=90	%
**Gender**		
Male	65	72.2
Female	25	27.8
**Age (range 18-73)**		
**Mean age ± standard deviation** 33.4 ± 11.6		
**Age group, years**		
18-24	21	23.3
25+	69	76.7
**Education level**		
Primary or lower	16	17.7
Secondary	32	35.6
Tertiary	42	46.7
**Occupation**		
Employed professional	42	46.7
Employed non-professional	34	37.8
In further study or seeking employment	14	15.5
**Residence**		
Rural	20	22.2
Semi-urban	51	56.7
Urban	19	21.2
**Smoking status**		
Waterpipe smoker	62	68.9
Non-smoker	28	31.1
**Ever noticed PHWs on WTPs**		
Yes	53	58.9
**Where**		
At cafés	17	32.1
At home	11	20.8
Buying tobacco	25	47.2

For peer review only - http://bmjopen.bmj.com/site/about/guidelines.xhtml

Interview guide *

Interview guide questions	Parts relevant to this article
**Introductory questions**<br>*In general, what do you think of waterpipe smoking?*<br>**Personal experiences of waterpipe smoking**<br>*Tell me about your waterpipe smoking, habits, duration, etc…Tell us more on how did you start smoking?*<br>*Could you describe the setting in which you smoked waterpipe last time?*	
**Awareness of pictorial health warnings**<br>*Have you ever noticed the pictorial health warnings on waterpipe tobacco packs?*<br>*Where did you see them? Can you tell us more about the setting?*<br>*How do they look like?*	√
**Positive and negative characteristics of the current/alternative set of PHW**<br>*Have you ever come across the warning on this pack before? What is your first impression about it?*<br>*What do you think/feel when you see it?*<br>*In your opinion, what is good and is bad about it? Why do you think so?*<br>*Do you think it is/would be effective? Why and how do you think so?*	√
**Reactions to placing PHW on the waterpipe device**<br>*What is your first impression about putting the warning in this spot?*<br>*What do you think/feel when you see there?*<br>*In your opinion, what is good and is bad about this spot? Why do you think so?*<br>*Do you think people will notice the PHW on this spot?*<br>*Would it be effective? Why and how do you think so?*	

***This guide contains core questions. Probing questions were used when necessary (examples include questions on salience, affective reactions, perceived harm, credibility)**

For peer review only

For peer review only - http://bmjopen.bmj.com/site/about/guidelines.xhtml

Table 3. Themes and subthemes identified from focus groups and in-depth interviews*

**Themes**	**Subthemes**	**Themes covered in this article**
General perceptions of waterpipe smoking	Factors contributing to WTS<br>Factors affecting WTS initiation and continuation<br>WTS setting<br>Patterns of WTS consumption<br>Social and cultural context of WTS<br>WTS compared to cigarette smoking<br>Tobacco product characteristics<br>Perceived health consequences<br>Reasons for quitting	
Views on pictorial health warning labels on waterpipe tobacco packs	General knowledge /awareness of PHWs on WTPs<br>Sources of awareness<br>Most salient/recalled labels, reasons<br>Cessation helpline<br>Perception of PHWs on WTPs motivating and discouraging factors related to viewing PHW labels	Partly
Perceptions of existing waterpipe tobacco warnings	General impressions, attitude (appeal, affective reactions, avoidance, wear out)<br>Positive and negative characteristics of design and content<br>Participants views of the most effective one in general<br>Possible effects on quitting and/or initiating WTS	√
Insights into novel waterpipe tobacco warnings	General impressions, attitude (appeal, affective reactions, avoidance)<br>Positive and negative characteristics of design and content<br>Participants views of the most effective one in general<br>Possible effects on quitting and/or initiating WTS	√
Avenues for development	Participant suggestions of improved label characteristics regarding content, design, location<br>Participant suggestions for other placement locations	Partly
Best location for inserting labels on the waterpipe device and accessories	Views behind choosing each location<br>Concerns about each location<br>Possible effects on quitting and/or initiating WTS	
Participant suggestions for reducing tobacco use in general and WTS in particular	Further strategies for improved regulations<br>Enforcing existing laws<br>Posting warnings in public and other places<br>Associate warnings with mass-media anti-tobacco campaigns, increase taxation and other suggestions	

*** Themes presented in this article were synthesized in respect to warning content and pack design and their perceived likely effect on uptake or cessation of waterpipe smoking, comparing existing versus novel warnings.**

For peer review only - http://bmjopen.bmj.com/site/about/guidelines.xhtml

Table 4. Selected participants' quotes representing views of the current and alternative pictorial health warning labels on waterpipe packs. Quotes are presented by gender, age group*, smoking status, and region of participants

Warning label content – perceived likely effect of existing and novel PHWs	
"The picture of a bent cigarette (i.e. representing sexual dysfunction) caught people's attention considerably…but the other warning…people got used to them after a while"	*(Male smoker, >25y, rural, FGD)*
"The most commonly known warning is that of the lungs"	*(Male smoker,>25y, semi-urban, FGD)*
"The picture is disgusting and frightening…"	*(Male non-smoker, <25y, rural, IDI)*
"Pictures are scary and unpleasant"	*(Male smoker, >25y, semi-urban, FGD)*
"The pictures make me sick…"	*(Male smoker, >25y, urban, IDI)*
"The warning shouldn't be that it causes cancer and that's it…that is just routine talk"	*(Male non-smoker, >25y, urban, FGD)*
"I just don't believe it…nobody reaches this stage"	*(Female smoker, <25y, urban, IDI)*
"I don't believe the warnings…I know smoking is harmful but not to that extent. This is unrealistic"	*(Male smoker, <25y, rural, FGD)*
"We want to see the normal lung beside the diseased one…but this one straight is not clear"	*(Male non-smoker, >25y, urban, FGD)*
"The pictures are not realistic, we never see such things in real life"	*(Male non-smoker, >25y, semi-urban, FGD)*
"The pictures are extremely exaggerated"	*(Male smoker, <25y, urban, IDI)*
"Is this a burnt piece of pizza? (face cancer warning)…this one won't be clear to illiterates…I didn't get it before reading the text beneath"	*(Male non-smoker, >25y, urban, FGD)*
"The picture is frightening and is very disgusting"	*(Male smoker, >25y, urban, IDI)*
"It can make me want to look closely to see what the illness shown is"	*(Female non-smoker, >25y, semi-urban, FGD)*
"For me, the most effective one is picture of teeth affection…it's very scary and upsetting. I see that content of the warning is much more important than size"	*(Male smoker, >25y, semi-urban, FGD)*
"With this shape (deformed teeth and gums) I may disturb my son when I come close to kiss him...now that will hurt me"	*(Male smoker, >25y, semi-urban, IDI)*
"The baby's picture may affect women more than me"	*(Male smoker, >25y, semi-urban, IDI)*
"If a pregnant woman saw this picture…she would fear for her unborn child to have anomalies"	*(Female smoker, >25y, semi-urban, FGD)*
"Explain the components of tobacco as glycerin and the hazards of each of them…tell them: try to smoke each component by itself and see what happens to you"	*(Male non-smoker, >25y, semi-urban, FGD)*
"Simplify the matter of quitting to people…especially those who have been smoking for a long time…"	*(Male nonsmoker, >25 y, rural, FGD)*
"If you talk to a smoker about their health…they wouldn't pay attention but there are other aspects as hazards to their children"	*(Male non-smoker,>25y, semi-urban, FGD)*
"The most effective thing that makes a smoker quit is that they themselves get ill or they see someone get ill"	*(Male smoker, >25y, semi-urban, IDI)*
" I saw on the internet a pack of cigarettes with a picture of a mother and	*(Female smoker, <25 y, urban, IDI)*

For peer review only - http://bmjopen.bmj.com/site/about/guidelines.xhtml

her child, and on opening it the mother is separated from him. This a new effective idea."	
"A pregnant woman or a mother may ask their smoker husband to stop"	(Female non-smoker, >25y, semi-urban, FGD)
"The only thing that can force me to stop smoking, even if I'm a heavy smoker is the baby affection"	(Female smoker, <25 y, urban, IDI)
"Warnings should target young people under 17 before they attempt smoking"	(Male non-smoker, >25y, semi-urban, FGD)
"Warnings concerning sexual dysfunction can affect young smokers"	(Male non-smoker,>25y, semi-urban, FGD)
"Focus on the damage that the family suffers…there are problems that can occur with the wife because of smoking…so smoking may endanger the family bonds"	(Male smoker, >25y, semi-urban, IDI)
"Pictures should be more realistic, and not only focus on severe side effects like cancers… like if warnings demonstrate just a fingernail affected due to smoking not all the hand, people will be more tempted to believe it"	(Male smoker, <25 year, rural, IDI)
Design Features of WTPs – perceived likely effect of existing and novel PHWs	
"The apples got my attention"	(Female smoker, >25y, semi-urban, IDI)
"After seeing this…(pointing to the fruit depicted on the pack)…it will be hard for me to link the idea with the damage in this picture"	(Male non-smoker, <25y, rural, FGD)
"Pictures of fruits are attractive and appetizing"	(Male smoker, >25y, semi-urban, FGD)
"If there is no flavored tobacco, I will quit"	(Male smoker, <25y, urban, IDI)
"I can't see any flavors on it…I want to hold it closely to know what did they put there instead…if shisha is served as it is…without any flavors?!...I'll quit"	(Male smoker, <25y, urban, FGD)
"If flavors were banned may be some will quit but warnings have no effect"	(Male smoker, <25y, urban, FGD)
"The pictures of fruits must be removed as they are appetizing"	(Male non-smoker, <25y, rural, IDI)
"The dark background makes the writings clearer"	(Male non-smoker,>25y, semi-urban, FGD)
"The alternative pack is clearer regarding the color, the picture and the way of writing"	(Male non-smoker, < 25y, rural, IDI)
"The black color makes the pack a lot chic"	(Male smoker, <25y, urban, FGD)
"The color of the package is dark and it conveys a sense of pessimism"	(Male smoker, >25y, rural, IDI)
"The bigger the picture, the better"	(Male smoker, <25y, semi-urban, FGD)
"The warning should be put on top because the pictures of fruits are drawing attention away from the warning"	(Female non-smoker, >25y, semi-urban, FGD)
"Oh! I didn't realize there was a quit line on the pack before…this dark background made some kind of contrast… the text now is way clearer for me to read"	(Female smoker, <25y, urban, IDI)
"I used to think about the hazards a lot when the pictures first appeared…then I got used to them…I don't pay them attention anymore"	(Male smoker, <25y, semi-urban, IDI)

For peer review only - http://bmjopen.bmj.com/site/about/guidelines.xhtml

"I have seen the warnings too often that they no longer frighten me"	*(Male smoker, <25y, rural, FGD)*
"The pictures are horrible but people got used to them, if a nonsmoker wished to try and saw the warnings they may change their mind but for those who are already smokers, the warnings make no difference	" *(Male non-smoker,>25y, semi-urban, FGD)*
"Warnings influence the non-smokers much more than smokers, as non-smokers usually find the picture more disgusting"	*(Female, non-smoker, >25y, semi-urban, FGD)*
"I avoid buying the picture of the tongue in particular (referring to the mouth cancer warning)"	*(Male smoker, <25y, semi-urban, FGD)*
"People can go around all day looking for a certain picture (the lungs)"	*(Male smoker, >25y, rural, IDI)*
"Vendors used to sell the pack with free stickers to cover the warnings"	*(Male smoker,>25y, semi-urban, FGD)*
"No one would buy a pack with such warning (means warning picturing effect of smoking on children)"	*(Female smoker, >25y, semi-urban, IDI)*
"If the warning on the pack looks bad, we tend to throw the external package away and put tobacco in another package or may be order a certain warning"	*(Male smoker, >25y, rural, FGD)*
"The alternative pack may make some smokers reduce smoking"	*(Male non-smoker, <25y, rural, FGD)*
"A beginner can be affected compared to a person who has been smoking for 15 years and nothing happened to them as a result of smoking"	*(Male non-smoker, <25y, rural, FGD)*
"Non-smokers will be more affected by it, smokers won't be much affected"	*(Female non-smoker, >25y, semi-urban, IDI)*
"It could affect those who want to start smoking, but the older smokers won't be affected much"	*(Male smoker, <25y, semi-urban, FGD)*

***The age group of participants was categorized as > or < 25 years, because we were interested in highlighting the views of young adults who may be at higher risk of experiencing WTS or maybe non-established WT users yet, versus older adults who might more likely have established a smoking or non-smoking behaviour.**

For peer review only - http://bmjopen.bmj.com/site/about/guidelines.xhtml

Reporting checklist for qualitative study.

Based on the SRQR guidelines.

Instructions to authors

Complete this checklist by entering the page numbers from your manuscript where readers will find each of the items listed below.

Your article may not currently address all the items on the checklist. Please modify your text to include the missing information. If you are certain that an item does not apply, please write "n/a" and provide a short explanation.

Upload your completed checklist as an extra file when you submit to a journal.

In your methods section, say that you used the SRQR reporting guidelines, and cite them as:

O'Brien BC, Harris IB, Beckman TJ, Reed DA, Cook DA. Standards for reporting qualitative research: a synthesis of recommendations. Acad Med. 2014;89(9):1245-1251.

		Reporting Item	Page Number
	#1	Concise description of the nature and topic of the study identifying the study as qualitative or indicating the approach (e.g. ethnography, grounded theory) or data collection methods (e.g. interview, focus group) is recommended	1,3, 8 & 9
	#2	Summary of the key elements of the study using the abstract format of the intended publication; typically includes background, purpose, methods, results and conclusions	3, 4
Problem formulation	#3	Description and signifcance of the problem / phenomenon studied: review of relevant theory and empirical work; problem statement	3, 5-9
Purpose or research question	#4	Purpose of the study and specific objectives or questions	3, 8
Qualitative approach	#5	Qualitative approach (e.g. ethnography,	8, 9

For peer review only

and research paradigm		grounded theory, case study, phenomenolgy, narrative research) and guiding theory if appropriate; identifying the research paradigm (e.g. postpositivist, constructivist / interpretivist) is also recommended; rationale. The rationale should briefly discuss the justification for choosing that theory, approach, method or technique rather than other options available; the assumptions and limitations implicit in those choices and how those choices influence study conclusions and transferability. As appropriate the rationale for several items might be discussed together.	See 5 and Supplementary file page 8 for more on limitations
Researcher characteristics and reflexivity	#6	Researchers' characteristics that may influence the research, including personal attributes, qualifications / experience, relationship with participants, assumptions and / or presuppositions; potential or actual interaction between researchers' characteristics and the research questions, approach, methods, results and / or transferability	11, 12 for reflexivity<br>Researchers are trained or experienced in qualitative methods. Word count limited detailing qualifications and other items listed here.
Context	#7	Setting / site and salient contextual factors; rationale	8, 9, 11
Sampling strategy	#8	How and why research participants, documents, or events were selected; criteria for deciding when no further sampling was necessary (e.g. sampling saturation); rationale	9, 10, 12
Ethical issues pertaining to human subjects	#9	Documentation of approval by an appropriate ethics review board and participant consent, or explanation for lack thereof; other confidentiality and data security issues	9, 11
Data collection	#10	Types of data collected; details of data	10, 11

For peer review only

methods		collection procedures including (as appropriate) start and stop dates of data collection and analysis, iterative process, triangulation of sources / methods, and modification of procedures in response to evolving study findings; rationale	
Data collection instruments and technologies	#11	Description of instruments (e.g. interview guides, questionnaires) and devices (e.g. audio recorders) used for data collection; if / how the instruments(s) changed over the course of the study	10, 11
Units of study	#12	Number and relevant characteristics of participants, documents, or events included in the study; level of participation (could be reported in results)	9, 10
Data processing	#13	Methods for processing data prior to and during analysis, including transcription, data entry, data management and security, verification of data integrity, data coding, and anonymisation / deidentification of excerpts	11
Data analysis	#14	Process by which inferences, themes, etc. were identified and developed, including the researchers involved in data analysis; usually references a specific paradigm or approach; rationale	11, 12
Techniques to enhance trustworthiness	#15	Techniques to enhance trustworthiness and credibility of data analysis (e.g. member checking, audit trail, triangulation); rationale	12
Syntheses and interpretation	#16	Main findings (e.g. interpretations, inferences, and themes); might include development of a theory or model, or integration with prior research or theory	12<br>See 9 for theory
Links to empirical data	#17	Evidence (e.g. quotes, field notes, text excerpts, photographs) to substantiate	13-17

For peer review only

		analytic findings	
Intergration with prior work, implications, transferability and contribution(s) to the field	#18	Short summary of main findings; explanation of how findings and conclusions connect to, support, elaborate on, or challenge conclusions of earlier scholarship; discussion of scope of application / generalizability; identification of unique contributions(s) to scholarship in a discipline or field	18-21<br>See also 3, 5 and Supplementary file page 8 for more on limitations
Limitations	#19	Trustworthiness and limitations of findings	5 and Supplementary file page 8 for more on limitations
Conflicts of interest	#20	Potential sources of influence of perceived influence on study conduct and conclusions; how these were managed	22
Funding	#21	Sources of funding and other support; role of funders in data collection, interpretation and reporting	22

The SRQR checklist is distributed with permission of Wolters Kluwer © 2014 by the Association of American Medical Colleges. This checklist can be completed online using https://www.goodreports.org/, a tool made by the EQUATOR Network in collaboration with Penelope.ai

For peer review only
